# The In-Feed Antibiotic Carbadox Induces Phage Gene Transcription in the Swine Gut Microbiome

**DOI:** 10.1128/mBio.00709-17

**Published:** 2017-08-08

**Authors:** Timothy A. Johnson, Torey Looft, Andrew J. Severin, Darrell O. Bayles, Daniel J. Nasko, K. Eric Wommack, Adina Howe, Heather K. Allen

**Affiliations:** a National Animal Disease Center, Agricultural Research Service, United States Department of Agriculture, Ames, Iowa, USA; b Genome Informatics Facility, Office of Biotechnology, Iowa State University, Ames, Iowa, USA; c Delaware Biotechnology Institute, University of Delaware, Newark, Delaware, USA; d Agricultural and Biosystems Engineering, Iowa State University, Ames, Iowa, USA; New York University

**Keywords:** agricultural antibiotics, carbadox, metatranscriptomics, prophage induction, swine

## Abstract

Carbadox is a quinoxaline-di-*N*-oxide antibiotic fed to over 40% of young pigs in the United States that has been shown to induce phage DNA transduction *in vitro*; however, the effects of carbadox on swine microbiome functions are poorly understood. We investigated the *in vivo* longitudinal effects of carbadox on swine gut microbial gene expression (fecal metatranscriptome) and phage population dynamics (fecal dsDNA viromes). Microbial metagenome, transcriptome, and virome sequences were annotated for taxonomic inference and gene function by using FIGfam (isofunctional homolog sequences) and SEED subsystems databases. When the beta diversities of microbial FIGfam annotations were compared, the control and carbadox communities were distinct 2 days after carbadox introduction. This effect was driven by carbadox-associated lower expression of FIGfams (*n* = 66) related to microbial respiration, carbohydrate utilization, and RNA metabolism (*q* < 0.1), suggesting bacteriostatic or bactericidal effects within certain populations. Interestingly, carbadox treatment caused greater expression of FIGfams related to all stages of the phage lytic cycle 2 days following the introduction of carbadox (*q* ≤0.07), suggesting the carbadox-mediated induction of prophages and phage DNA recombination. These effects were diminished by 7 days of continuous carbadox in the feed, suggesting an acute impact. Additionally, the viromes included a few genes that encoded resistance to tetracycline, aminoglycoside, and beta-lactam antibiotics but these did not change in frequency over time or with treatment. The results show decreased bacterial growth and metabolism, prophage induction, and potential transduction of bacterial fitness genes in swine gut bacterial communities as a result of carbadox administration.

## IMPORTANCE

FDA regulations on agricultural antibiotic use have focused on antibiotics that are important for human medicine. Carbadox is an antibiotic not used in humans but frequently used on U.S. pig farms. It is important to study possible side effects of carbadox use because it has been shown to promote bacterial evolution, which could indirectly impact antibiotic resistance in bacteria of clinical importance. Interestingly, the present study shows greater prophage gene expression in feces from carbadox-fed animals than in feces from nonmedicated animals 2 days after the initiation of in-feed carbadox treatment. Importantly, the phage genetic material isolated in this study contained genes that could provide resistance to antibiotics that are important in human medicine, indicating that human-relevant antibiotic resistance genes are mobile between bacteria via phages. This study highlights the collateral effects of antibiotics and demonstrates the need to consider diverse antibiotic effects whenever antibiotics are being used or new regulations are considered.

## INTRODUCTION

Antibiotics are fed to livestock and poultry to treat and prevent disease. Since January 2017, antibiotics deemed important to human medicine were disallowed as animal growth promoters in the United States under the regulation of the Veterinary Feed Directive (VFD) (1). Antibiotic use in both humans and animals selects for the evolution and dissemination of antibiotic resistance genes through mobile genetic elements such as phages and plasmids (2). Once they are incorporated within the genomes of human-pathogenic bacteria, these antibiotic resistance genes impede disease treatment (3). In addition to an increase in antibiotic resistance, antibiotics at subinhibitory concentrations alter bacterial physiology, including gene expression (4, 5), and the frequent use of antibiotics in animal agriculture has contraindicated effects on the gut microbiome, including altered microbiota composition (6), pathogen colonization susceptibility (7), co-selection for unintended antibiotic resistance genes (8, 9), increased abundance of mobile genetic elements (10), and increased horizontal transfer of antibiotic resistance genes via conjugation and transduction (11). These collateral effects of antibiotic use are important to consider when devising policies for improving antibiotic stewardship.

One antibiotic that has significant collateral effects on bacterial evolution is carbadox. Carbadox is a quinoxaline-di-*N*-oxide antibiotic allowed in United States swine husbandry for growth promotion and treatment and suppression of swine dysentery during weaning (12). Carbadox (at 5 ppm) is genotoxic (13) and mutagenic (14), and yet it has no analogue in human medicine so its use is not slated to be reformed by the current VFD guidelines. In addition to its mutagenic properties, *in vitro* studies have shown that a carbadox concentration below the minimal inhibitory concentration induces the SOS pathway in *Escherichia coli* and *Salmonella enterica* serovar Typhimurium (15) and kills *S*. Typhimurium by induction of prophage, followed by phage-mediated bacterial lysis rather than by direct action on bacterial physiology by the molecule (16). Phage induction, as understood in *E. coli*, occurs because of DNA damage-induced initiation of the SOS response, which begins with the expression of *recA*. RecA binds and cleaves the SOS suppressor LexA to allow expression of SOS response genes, as well as LexA-suppressed phage genes (17). Cultures of *Brachyspira hyodysenteriae* treated with carbadox showed increased expression of the *hvp38* gene (encodes a known phage major capsid protein) and the production of VSH-1 DNA and VSH-1 particles, a prophage-like gene transfer agent (18). Additionally, carbadox facilitated the generalized transduction of phage and chromosomal DNA *in vitro* (16). Carbadox-induced bacteriophage have been shown to horizontally transfer genes that can confer resistance to chloramphenicol (16, 18), tetracycline (16), carbenicillin (16), and tylosin (18) between bacterial strains. Unlike most antimicrobials, carbadox, mitomycin C, metronidazole, and hydrogen peroxide cause DNA damage, which causes bacterial stress responses and the induction of prophages within the bacterial genome (18). Nearly 50% of all reported bacterial genomes contain lysogenic prophage sequences (19); thus, an antibiotic that induces prophage, like carbadox, could have profound impacts on many bacterial populations within a natural bacterial community.

The goals of this study were to further define the breadth of carbadox-mediated impacts on the swine microbiome and to investigate the differential expression of bacterial gene families that may reveal global impacts on swine gut bacterial communities. Previous research has shown a carbadox-mediated increase in phage-encoded phage integrases within swine gut microbial metagenomes, which could be a proxy for prophage induction (20). The present study improves upon the previous work by sequencing viromes from individual animals and correlating them with microbial metagenomic, metatranscriptomic, and 16S rRNA gene-based bacterial diversity analyses. Here we show differential gene expression suggestive of prophage induction in the swine gut microbial metagenome 2 days following carbadox administration and additionally demonstrate effects of carbadox on bacterial metabolism and respiration.

## RESULTS

### Metagenomic sequencing of swine gut microbial communities.

To construct a single reference assembly, all of the sequence data from 47 samples (microbial metagenome, metatranscriptomes, and virome) were incorporated into one co-assembly. Although the primary sequencing target in this study was metatranscriptomic mRNA, inclusion of longer metagenomic sequences (because of the sequencing platform used) resulted in the assembly of longer contigs. Total sequencing output (metatranscriptome, 250 Gbp; microbial metagenome, 5 Gbp; virome, 1.3 Gbp) resulted in an assembly (605 Mbp) composed of 572,839 contigs, the longest of which was 125 kb, and the N50 (i.e., median) contig length was 1.5 kb. On average, 44% of the metatranscriptome and 31% of the phage sequences mapped to the composite assembly. The exclusive phage assembly (85 Mbp) was composed of 51,640 contigs, the longest of which was 227,766 bp, and the N50 contig length was 2 kb (see Table S2 in the supplemental material).

### Phylogenetic and functional composition of the swine gut metatranscriptome.

**FIG 1:**
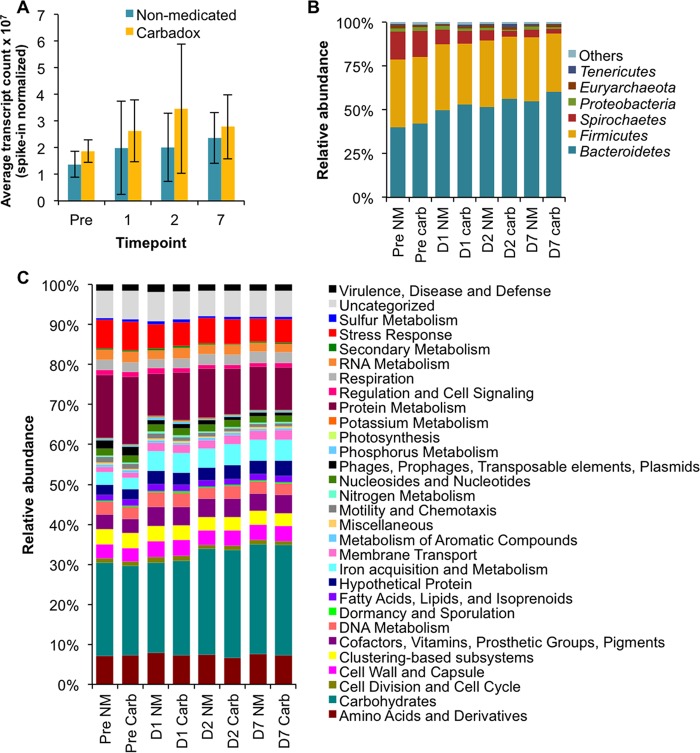
Total composition of microbial metatranscriptomes. (A) Average total number of transcripts obtained from each sample normalized by the number of artificial-RNA-spiked sequences obtained per sample. Error bars indicate the standard error of the six replicates of each treatment. (B, C) Relative abundance of annotated ORFs assigned to taxa (B) and SEED subsystem assignments of FIGfams (C). The Pre time point was 5 days prior to carbadox addition to feed. NM, nonmedicated; carb, carbadox; D, day.

The metatranscriptomic sequences were mapped onto the composite assembly, counted, and normalized by the sequencing yield of spiked-in control RNA. The total normalized RNA counts per sample were not statistically significantly different because of treatment (*t* test, *P* > 0.05), and variance within treatment groups was high (Fig. 1A). Phylogenetic inference of the annotated open reading frames (ORFs) showed that the phylum-level taxonomic patterns were similar between treatment groups. The phylum-level taxonomic inferences of the metatranscriptomes were not statistically significantly different (*q* [false discovery rate] of >0.05) between treatment groups and over time. The metatranscriptome was dominated by *Bacteroidetes* (40 to 60% of the community) and *Firmicutes* (40%), with a minor contribution from *Spirochaetes* (Fig. 1B). There was a trend for *Bacteroidetes* transcripts to increase with carbadox treatment (*q* = 0.17). The Shannon diversity of taxonomic assignments tended to decrease with carbadox treatment (Fig. S1) and was statistically significantly decreased on day 1 (*t* test, *P* = 0.04).

10.1128/mBio.00709-17.2 FIG S1  Shown are the Shannon (A) and Simpson (B) diversity indices based on the taxonomic assignment of the sequences of the metatranscriptome libraries. The asterisk indicates a statistically significant difference in Shannon diversity (*t* test, *P* < 0.05). med, medicated; d, day. Download FIG S1, TIF file, 8 MB. Copyright © 2017 Johnson et al. 2017 Johnson et al. This content is distributed under the terms of the Creative Commons Attribution 4.0 International license.

**FIG 2:**
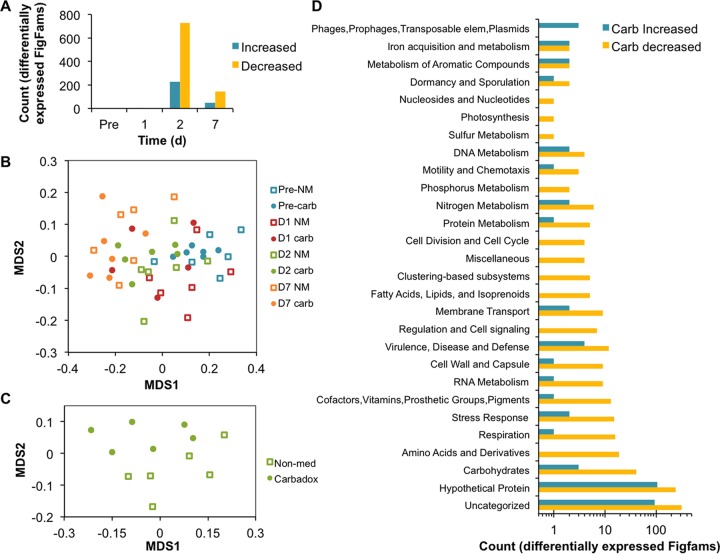
Differential-expression analysis of ORFs annotated with the FIGfams database. (A) Total number of differentially expressed FIGfams at each time point in response to in-feed carbadox. FIGfams with higher abundance in the carbadox treatment group than in the nonmedicated group were considered carbadox (Carb) increased, while FIGfams with lower abundance in the carbadox treatment group than in the nonmedicated group were considered carbadox decreased. (B) NMDS ordination of metatranscriptome FIGfam counts. Sample shifts exhibit a time gradient, but no clear clustering because of treatment was observed. Stress for the analysis is fairly high (0.16). (C) NMDS ordination of sample FIGfam counts at day 2. An ordination of data from each day was completed separately to obtain more acceptable stress values, and only on day 2 was there a statistically significant separation of the samples by treatment (*P* < 0.1). The stress for this ordination was 0.10. (D) Number of differentially expressed FIGfams binned by their SEED subsystem assignments. A large number of ORFs were assigned to a FIGfam but not assigned to a SEED subsystem and therefore were binned in the uncategorized group. The members of the uncategorized bin were manually inspected (see Text S1) and included in subsequent subsystem-specific summaries (Fig. 3; Fig. S2). elem, elements.

The mapped metatranscriptomic sequences were also analyzed for function by using the hierarchical classification of function by SEED subsystems. The most abundant broad functional subsystems were carbohydrates and protein metabolism, and the abundance of all of the subsystems detected was consistent between treatment groups and through the sampling time period (Fig. 1C). Fine-scale resolution of SEED subsystem functions was achieved by using FIGfam annotations, which represent isofunctional homologs. No differentially expressed FIGfams were observed in the treatment groups prior to antibiotic administration. Differential expression of FIGfams (*q* of <0.1) peaked 2 days after the introduction of carbadox, when nearly 1,000 FIGfams were differentially expressed (730 more abundant in nonmedicated animals and 227 more abundant in carbadox-fed animals), in contrast to only 4 and 197 differentially expressed FIGfams (*q* of <0.1) on days 1 and 7, respectively (Fig. 2A). This treatment-based divergence at the day 2 time point is reflected in the distribution of the beta diversity of the functional assignments. The nonmedicated and carbadox treatment groups clustered separately (goodness of fit *P* = 0.054, based on 10^6^ permutations) on day 2 (Fig. 2C) but not on any of the other days. The beta diversity of the FIGfam counts in the carbadox and nonmedicated control groups both shifted over the time frame of the experiment (nonmetric multidimensional scaling [NMDS] analysis [Bray-Curtis diversity measure, Fig. 2B, stress = 0.16]).

Differentially expressed FIGfams were binned according to SEED subsystem (functional categories) to identify those bacterial functional systems most impacted by carbadox treatment on day 2. Interestingly, only the phages, prophages, transposable elements, and plasmids subsystem had more upregulated FIGfams than downregulated FIGfams in response to carbadox. Every other SEED subsystem was downregulated in medicated animals. The subsystems with the most downregulated FIGfams in carbadox-fed animals were carbohydrates, amino acids and derivatives, respiration, and stress response (Fig. 2D; see Text S1 for more information). Principal among these was the carbohydrates subsystem (Fig. S2A). Central metabolism pathways, especially components of pyruvate metabolism and the pentose phosphate pathway, were downregulated. Coupled with the decreased expression of metabolic pathways was the decreased expression of respiration genes (Fig. 2D; see Text S1 for more information). Specifically, genes involved in electron-accepting and electron-donating reactions were largely downregulated. Although these significant effects were observed in bacterial growth and metabolism, evidence of a bacterial stress response due to carbadox perturbation was limited. One day after carbadox administration, there were no differentially expressed stress response FIGfams, and 2 days following carbadox introduction, when carbadox administration manifested its greatest effect on microbial gene expression, the bacterial stress response was suppressed (Fig. S2C).

10.1128/mBio.00709-17.1 TEXT S1  Supplemental materials and methods, results, and references. Download TEXT S1, DOCX file, 0.03 MB. Copyright © 2017 Johnson et al. 2017 Johnson et al. This content is distributed under the terms of the Creative Commons Attribution 4.0 International license.

10.1128/mBio.00709-17.3 FIG S2  Fold changes in FIGfam expression within the carbohydrates (A), respiration (B), and stress response and DNA repair (C) subsystems because of carbadox. FIGfams with a positive log_2_-fold change were more abundant in carbadox-treated animals, while those with a negative log_2_-fold change were more abundant in nonmedicated animals. FIGfams followed by an asterisk were manually assigned to the subsystem indicated on the basis of the FIGfam name (e.g., mannan endo-1,4-beta-mannosidase). Different-color bars indicate functional categories of FIGfams. Download FIG S2, JPG file, 0.6 MB. Copyright © 2017 Johnson et al. 2017 Johnson et al. This content is distributed under the terms of the Creative Commons Attribution 4.0 International license.

10.1128/mBio.00709-17.4 FIG S3  Differentially expressed SOS pathway-related sequences in the metatranscriptome on day 2. (A) ORFs that were annotated as *recA*, *lexA*, *ruvA*, *ruvB*, *uvrA*, *uvrB*, or *uvrC* were then binned according to their taxonomic assignment. Genera with an SOS pathway gene differentially expressed (*q* of <0.10) are shown here. (B) Sequences assigned to the *recA* FIGfam were individually compared for differential expression (*q* of <0.05). Taxonomic assignment is shown as the label. Since this analysis was performed on individual sequences, multiple sequences were assigned to the same genus. Non-med, nonmedicated. Download FIG S3, TIF file, 11.2 MB. Copyright © 2017 Johnson et al. 2017 Johnson et al. This content is distributed under the terms of the Creative Commons Attribution 4.0 International license.

### Carbadox caused differential expression of phage genes.

**FIG 3:**
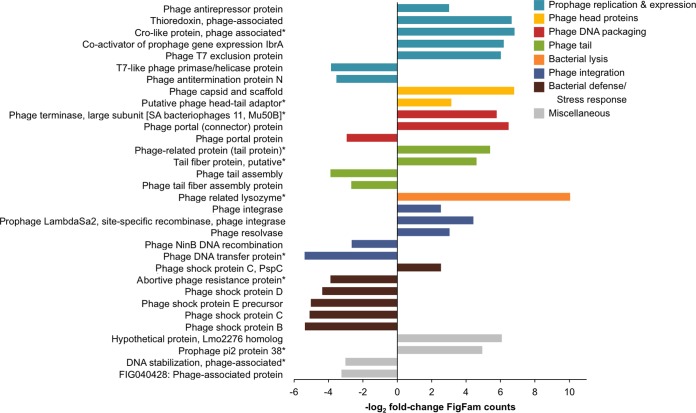
Fold changes in phage-related FIGfam expression 2 days following carbadox administration. FIGfams with a positive log_2_-fold change were more abundant in the carbadox-treated animals, while those with a negative log_2_-fold change were more abundant in the nonmedicated animals. FIGfams followed by an asterisk were manually assigned to the phage subsystem on the basis of the FIGfam name (e.g., phage-related lysozyme). Different-color bars indicate functional categories of FIGfams.

Among the 957 differentially expressed FIGfams on day 2, 32 were related to phage (18 were upregulated and 14 were downregulated with carbadox) (Fig. 3). Many of these differentially expressed genes are related to the lytic phage cycle. The induction of the lytic phage cycle is a multistep pathway involving prophage gene expression, phage capsid synthesis, bacterial lysis, and phage recombinases in new bacterial lysogens. We observed gene expression shifts 2 days following carbadox exposure that indicate phage lytic cycle genes were activated within the bacterial populations of the swine gut. FIGfams were differentially expressed that could facilitate the induction of the phage lytic cycle, for example, the lysogenic cycle repressor *cro* (overexpressed 112-fold) and *ibrA*, a coactivator of prophage gene expression (overexpressed 73-fold). FIGfams specific to phage capsid synthesis were overexpressed because of carbadox, including phage capsid and scaffold proteins (enriched nearly 100-fold) and putative head-tail adapter proteins. Gene products important for phage DNA packaging were also upregulated; the nascent phage chromosome is translocated to and inserted into the phage capsid by both phage terminase and phage portal proteins, which were 52- and 91-fold enriched, respectively. The terminal steps in the phage lytic cycle include bacterial lysis and reinfection of new lysogens. Phage lysozyme was the most differentially expressed phage FIGfam (>1,000-fold enriched with carbadox treatment) and would likely facilitate bacterial lysis. Protein families involved with phage DNA integration into the bacterial chromosome (phage integrase, prophage site-specific recombinase, and phage resolvase) were overexpressed, on average, 12-fold. Interestingly, of the phage-related FIGfams (Fig. 3) for which a taxonomy could be predicted (*n* = 18), all that were overexpressed were predicted to be encoded by diverse members of the Gram-positive phylum *Firmicutes*. Thus, this phylum might be the most strongly impacted by phage induction.

Even though the expression of most phage lytic cycle-related FIGfams was increased because of carbadox on day 2, an important set of phage-related FIGfams that mainly play a role in bacterial defense and response to phage was underexpressed. Four phage shock proteins (PSPs; bacterial stress response to invading phage [21]) were underexpressed (approximately 25-fold). To further understand the bacterial defense against phages, CRISPRs (clustered regularly interspaced short palindromic repeats) were investigated because they aid bacteria in the recognition and destruction of incoming phage DNA. The results showed 372 CRISPR arrays in the composite assembly. Although 14% of the spacers were validated by identical full-length matches in the virome, no differential expression of CRISPRs because of carbadox treatment was detected in the metatranscriptome. Taken together, the differentially expressed phage genes show the impact of carbadox on phages within the greater intestinal microbiota soon after carbadox exposure. Carbadox appears to induce the production of phages, and the bacterial host defense and response to bacteriophage appeared to be lowered.

### Phylogenetic and functional composition of the swine virome.

**FIG 4:**
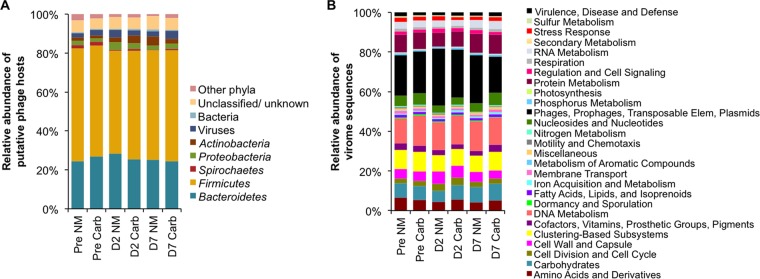
Total composition of viromes based on virome annotations. (A) Relative abundance of virome sequences based on putative taxonomic assignment based on the top BLASTP match by VIROME. (B) SEED subsystem functional categories of virome sequences. The Pre time point was 5 days prior to carbadox use in feed. NM, nonmedicated; Carb, carbadox; Elem, elements.

Interestingly, in agreement with the increased expression of *Firmicutes* prophage genes from the metatranscriptome, the ORFs obtained from the virome were predominantly (60% of the virome) homologous to *Firmicutes* phage, while only 25% of the virome was homologous to *Bacteroidetes* phage (Fig. 4). *Prevotella*, the major member of the *Bacteroidetes* phylum in the swine microbiome, accounted for only about 10% of the predicted hosts of the phage sequences, which is interesting because a previous analysis of the 16S rRNA gene sequences from these samples showed that *Prevotella* bacteria composed about 50% of the bacterial community (22) and the metatranscriptome data show that they are an active population (composing 30 to 50% of the transcripts [Fig. S5A]), suggesting that *Prevotella* bacteria have fewer prophage in their genome or the prophage they do have might not be induced by carbadox. Other phage hosts classified at the genus level are disproportionately impacted. The virome was enriched with sequences homologous to *Clostridium* and *Bacteroides*, representing about 25% of the virome, relative to the abundance of these two genera in the metatranscriptome (15%) and 16S rRNA amplicon (1%) data sets (22).

Phages make up only a small but diverse subset of the bacterial pangenome. Approximately 20% of the swine gut virome encoded phage-specific functions, but the virome also contained annotations that represent broad functional potential. In fact, nearly all of the SEED subsystems from the metatranscriptome were represented in the viromes, which also suggests that a broad set of genes can be transferred via transduction. Relative to the microbial metatranscriptome, functions related to carbohydrates, respiration, stress response, and protein metabolism were underrepresented in the viromes. Functions related to DNA metabolism, nucleosides and nucleotides, and phage, prophage, transposable elements, and plasmids were overrepresented.

### Antibiotic resistance genes in the virome and the metatranscriptome.

**FIG 5:**
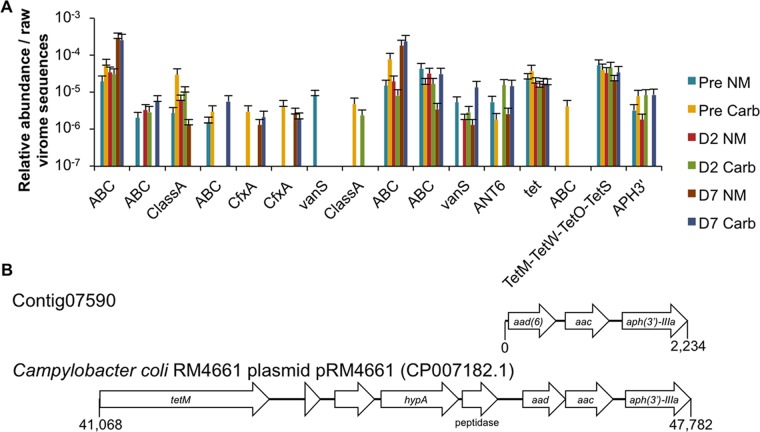
Antibiotic resistance genes observed in virome sequences. (A) Counts of antibiotic resistance genes as annotated by Resfams. Entries with identical labels indicate different genes that belong to the same resistance gene family, as defined by Resfams. Antibiotic resistance gene counts are shown to demonstrate their abundance in the virome, but there were no statistically significant differences in the abundance of resistance genes in time-matched samples. The Pre time point was 5 days prior to carbadox use in feed. NM, nonmedicated; Carb, carbadox; D, day. (B) ORF map of one phage contig (Contig07590) containing three aminoglycoside resistance genes and its alignment with the pRM4661 plasmid from *C. coli* (NCBI GenBank accession number CP007182.1). Contig 07590 was 99% identical to the nucleotide sequence of the pRM4661 plasmid over 93% of its length. ABC, ATP-binding cassette transporters; ClassA, class A beta lactamase.

Because of the possibility of phage-mediated horizontal gene transfer between bacterial populations, a phenomenon that has been observed *in vitro* in the presence of carbadox (16, 18), antibiotic resistance genes included in the swine gut virome would be of particular interest. A set of genes potentially conferring resistance to multiple classes of antibiotics, as well as efflux pumps, were identified within the virome libraries (Table S3) and the microbial metatranscriptome (Fig. S4). With the low number of sequences obtained from the viromes, no statistically significant differences in the relative abundance of antibiotic resistance in the phage sequences due to carbadox treatment were detected (Fig. 5A). Nevertheless, two interesting results were revealed by the investigation of contigs containing resistance genes, regardless of carbadox treatment. First, a resistance gene was frequently the only gene in the contig, potentially because of flanking repetitive elements or low sequence coverage impeding assembly to the larger genetic context. This is important because resistance genes could have been mobilized by mobile genetic elements to prophage islands for subsequent horizontal transfer by lytic phage induction and later invasion of new bacterial hosts. Indeed, the transcription of mobile genetic elements that could then allow the mobilization of DNA within a bacterial cell was impacted both positively and negatively by carbadox: conjugative transfer proteins TrbE and TrbG, the transposase IS*605* OrfB family, and IS*Sod11* transposase were all overexpressed with carbadox, while IS*6770* transposase, Tn*7*-like transposase TnsA, and IS*1478* transposase were downregulated. Second, of the 14 contigs that contained an antibiotic resistance gene, 1 phage contig contained multiple antibiotic resistance genes, i.e., for an aminoglycoside 6-adenylyltansferase, a streptothricin acetyltransferase, and an aminoglycoside phosphotransferase (Fig. 5B). The aminoglycoside phosphotransferase (*aph3*′) gene on this contig tended to be more abundant in carbadox-treated animals (Fig. 5A), but this difference was not statistically significant. This contig is 99% identical over 93% of its length to the pRM4661 plasmid from *Campylobacter coli* (accession no. CP007182.1), demonstrating overlap between the resistomes of phages and plasmids belonging to members of the gut microbiota.

10.1128/mBio.00709-17.5 FIG S4  Counts of differentially expressed (*q* of <0.05) antibiotic resistance genes in the metatranscriptome as annotated by Resfams. Download FIG S4, TIF file, 11.2 MB. Copyright © 2017 Johnson et al. 2017 Johnson et al. This content is distributed under the terms of the Creative Commons Attribution 4.0 International license.

10.1128/mBio.00709-17.6 FIG S5  Relative abundance of genus level taxa in the metatranscriptome (A) and virome (B) sequences based on putative taxonomic assignment of the top BLASTP match by VIROME. The first nine genera are color matched in both panels A and B. (C) NMDS ordination of the virome based on taxonomic assignments. NM, nonmedicated; Carb, carbadox; D, day. Download FIG S5, TIF file, 8 MB. Copyright © 2017 Johnson et al. 2017 Johnson et al. This content is distributed under the terms of the Creative Commons Attribution 4.0 International license.

In the metatranscriptome, the expression of most resistance genes was decreased with carbadox administration; however, four genes enriched by carbadox administration were those for an aminoglycoside acetyltransferase (*aac3*) (20-fold enriched), a class A beta-lactamase (10-fold enriched), and two ATP-binding cassette (ABC) efflux pumps (each about 10-fold enriched). Resistance to carbadox has been defined to be gained by an efflux pump, encoded by *oqxAB* (23), but these genes were not detected in our data set. Our results show two ABC efflux pumps with increased expression, while genes conferring resistance to other antibiotics are generally underexpressed (Fig. S4).

### FIGfams differentially expressed on days 1 and 7.

Because of the distinct shift in gene expression 2 days following carbadox introduction, we investigated whether any related shifts were observed on days 1 and 7 to estimate the time frame of the disturbance. On day 1, only four FIGfams were differentially expressed (all upregulated) and two were phage proteins (Fig. S6A). On day 7, 147 FIGfams were differentially expressed; 7 of these were phage-related FIGfams, and 5 were downregulated (three structural proteins and two nucleic acid polymerases), while 2 were upregulated (Fig. S6B). Additionally, FIGfams related to carbohydrate, respiration, and amino acid subsystems were mostly downregulated, as they were on day 2 but to a lesser extent on day 7 (Fig. S6C). Again, FIGfams related to pyruvate and acetoin metabolism, cytochromes, and hydrogenases were downregulated as they were on day 2. These results are a stark contrast to the nearly 1,000 genes that are differentially expressed at day 2, illustrating the temporal dynamics of the effect of continuous in-feed carbadox on the gut microbiota.

10.1128/mBio.00709-17.7 FIG S6  Differentially expressed FIGfams on days 1 and 7 following carbadox (Carb) administration in feed. (A) Fold changes in all differentially expressed FIGfams (*n* = 4) on day 1 following carbadox addition to feed. There were no FIGfams with higher expression in nonmedicated animals than in carbadox-feed animals. (B) Differentially expressed FIGfams assigned to the phage subsystem. As opposed to the differential expression of phage-related FIGfams on day 2, most of these FIGfams are underexpressed with carbadox. (C) Number of differentially expressed FIGfams binned by their SEED subsystem assignments. FIGfam and SEED annotations were done indepentantly. Download FIG S6, JPG file, 0.3 MB. Copyright © 2017 Johnson et al. 2017 Johnson et al. This content is distributed under the terms of the Creative Commons Attribution 4.0 International license.

## DISCUSSION

We investigated the effects of an in-feed antibiotic, carbadox, on phage communities and bacterial gene expression within the swine gut. In the United States, the VFD (1), effective January 2017, does not regulate carbadox because it has no analogue in human medicine; however, carbadox is under consideration for withdrawal from use in the United States because of a joint recommendation by the FAO/WHO Food Standards Programme (24) because of its potential mutagenic properties. Although the future use of carbadox in agriculture is uncertain, we show that a collateral effect of the feed additive carbadox is upregulation of genes related to all stages of the phage lytic cycle during the same perturbation time frame in which shifts in bacterial composition were also observed. Induction of the phage lytic cycle could alter bacterial community composition on the basis of the prophage content (19) and stability (25) of specific bacterial and phage populations and might facilitate antibiotic resistance gene transfer by transduction.

When considering all of the effects of carbadox, the genes that were overexpressed show that a multistep induction of the phage lytic cycle is one possible response. The phage lytic cycle was the only functional pathway that showed an overall increase in gene expression with carbadox treatment. This induction pathway is initiated by selective overexpression of phage genes and repression of bacterial genes. Differentially expressed genes in the swine gut microbial metagenome play a role in lytic phage gene replication and expression, i.e., *cro* (26), phage antirepressor genes (27), and the thioredoxin gene (28). After phage genes are expressed and transcribed, bacteriophage assembly (29) is initiated. Genes enriched under carbadox treatment play a role in phage assembly; the phage capsid and phage head-tail adaptor would aid in initiating phage construction, and DNA packaging into the capsid (30) would be aided by both phage terminase and portal proteins (31). Phage assembly is completed by phage tail assembly. In our data set, on day 2, expression of genes encoding phage tail proteins was enriched while expression of phage tail assembly proteins was decreased with carbadox. Genes involved in lysogen invasion and phage DNA recombination, the terminal step of the phage lytic cycle, were also enriched in our data set, including phage lysozyme (bacterial lysis [32]), phage integrase and prophage site-specific recombinase (DNA recombination in new bacterial hosts), and phage resolvase (resolves Holliday junctions during phage recombination [33]). A previous study measured an increase in DNA encoding phage integrase in the swine gut microbial metagenome (20). By interrogating the metatranscriptome, we observed a more detailed impact of carbadox on prophage with the overexpression of many lytic cycle genes in the swine gut microbiome.

Others have shown transcriptomic changes during prophage induction *in vitro* with single strains that support our findings. Hare et al. observed many genes with differential expression in *Acinetobacter baumannii* ATCC 17978 and *Acinetobacter baylyi* ADP1 because of prophage induction by mitomycin C. A subset of those genes overlaps the differentially expressed genes in our data set, i.e., *cro* and genes for a putative helicase, a major capsid protein, a head-tail adapter protein, a phage terminase, phage tail proteins, and a phage-related lysozyme (34). A second study examined gene expression throughout the phage infection process of *Yersinia enterocolitica* by φR1-37. In that model, the investigators showed that PSPs were overexpressed late in the infection process (35), which contrasts with their underexpression in our data set. However, the only perturbation in the study by Leskinen et al. was the addition of viral particles, which is sufficient to induce a PSP response (36). Although the cause for the suppression of the *psp* operon in the presence of carbadox is unknown, the downregulation is potentially due to general growth inhibition by carbadox, the timing of our samples capturing the early rather than the late lytic cycle, or an energy conservation strategy of the bacterial community since other stress response pathways are also underexpressed.

An important aspect of phage induction is the potential for transduction, which is phage-mediated gene transfer. Antibiotic resistance genes, including genes that confer resistance to tetracycline, beta-lactams, and aminoglycosides (as well as efflux pumps), were located in the swine gut virome, as well as one set of colocalized antibiotic resistance genes. By inducing phages and their subsequent infection of new bacterial hosts, phage genes (including antibiotic resistance genes) could be transferred to the bacterial metagenome, and this has been shown to occur *in vitro* (16, 18). Even though these resistance genes are not relatively more abundant in carbadox-fed swine gut viromes (determination of absolute abundance was not attempted), an increase in phage transduction promotes the exchange of bacterial and phage DNA, including antibiotic resistance genes, which is detrimental at any frequency (16, 18). The risk of horizontal transfer of an antibiotic resistance gene between bacterial populations is greatly amplified if it is carried by any mobile genetic element (37), including a bacteriophage.

Recently, Enault et al. cautioned that phages rarely contain antibiotic resistance genes (38) and that prediction of antibiotic resistance genes in sequenced viromes may overestimate their abundance. In known phage genomes, they identified two true antibiotic resistance genes among 121,506 viral proteins (0.0016%). We have predicted antibiotic resistance genes at a marginally higher rate (14/141,701 ORFs [0.010%] in the virome assembly). We predicted antibiotic resistance genes by using the Resfams database, which was shown to be relatively stringent in assigning antibiotic resistance genes and accurately predicted the number of viral antibiotic resistance genes in fecal material (38). In addition, there is a precedent for antibiotic resistance genes to be present in the virome, especially considering that carbadox is known to induce the generalized transduction of antibiotic resistance genes (16). Finally, CsCl gradients were used to purify viral particles from each sample, and this method produces more purified viral particles than other methods (39). These protocols reduce the possibility that antibiotic resistance genes from nonviral DNA sources contaminated our virome libraries. Thus, it is likely that these antibiotic resistance genes were packaged within viral particles.

By 16S rRNA gene sequence analysis of the samples used in the present study, *Prevotella* was shown to maintain the same population size both before and 1 week after carbadox administration, concurrent with a decrease in the total bacterial community size (22). This stability of the *Prevotella* population does not appear to be due to a lack of prophages. A study by Touchon et al. showed an average prophage content of 1.25 per *Prevotella* genome (19). However, relatively few of our virome sequences were homologous to *Prevotella* phage. This could be in part because host assignment of phages based on the taxonomy of homologous sequences lacks precision. Preliminarily, prophage induction seems to depend on prophage instability (25) in the presence of carbadox, but further analysis of phages within a taxonomic group of interest is required.

In bacteria, the best-understood pathway of prophage induction is DNA damage-mediated induction of the SOS response, which is manifested by increased expression of *recA*. RecA binds to and removes the repressor *lexA* from SOS pathway genes, as well as phage genes (17). Because carbadox causes DNA damage, we would expect that genes related to DNA repair and stress response would show increased transcription in samples from carbadox-fed pigs. The lack of change in the SOS response or even an oxidative stress response with carbadox treatment that we observed in our data set has many potential explanations. Although an SOS response to DNA damage is a canonical response in *E. coli* (17, 40), it appears that the SOS response to DNA damage is subtle, varied, and time dependent between species. *In vitro* experiments show that *recA* is induced only 5- to 10-fold in both *E. coli* (41, 42) and *Acinetobacter* (34) species. Furthermore, expression of SOS pathway genes is not uniformly induced, even in closely related species (43), and the SOS response is rapid, with maximal RecA production shown to occur within 90 min of exposure to mitomycin C (44). It is therefore possible that our sample collection was too late (24 h) and the complex bacterial community was too heterogeneous (the stratified gut environment) to capture a general bacterial SOS response to carbadox. It is also possible that some phage are induced in the absence of an SOS response; one such induction mechanism is phage antirepressors (27), which were moderately overexpressed because of carbadox in our data set. Despite varied SOS responses to DNA damage in multiple experiments, prophage induction seems to be a longer sustained response to DNA damage (17) among many taxa (18, 35, 45–47).

No previous metatranscriptome experiments have been conducted in the presence of carbadox; however, microbial metatranscriptomes have been reported twice in swine. Poroyko et al. sequenced cecal metatranscriptomes from four formula-fed and four sow-fed piglets that were 21 days old. Functional gene transcription was very similar in the two treatment groups. Non-rRNA transcripts (a total of 139,114) related to carbohydrate, protein metabolism, stress response, and amino acids were the most abundant SEED subsystems detected in their study (48). A second study included six proximal-colon swine metatranscriptomes (a total of 20,087,837 sequences), but the researchers only considered antibiotic resistance and secondary metabolism genes in their analysis (49). The work in our study represents the most extensive swine metatranscriptome sequencing effort to date, in terms of both the number of animal samples (*n* = 47) and the total number of sequences (1.8 billion). It was this sequencing effort that allowed us to uncover the induction of prophage since these are a minor portion of the bacterial metatranscriptome.

In our data set, metabolism and respiration genes were underexpressed, suggesting that the metabolic state of the microbiota was significantly affected by carbadox and the ability of the microbiota to use carbon sources and their subsequent energy production were hampered. Antibiotics have been shown to alter the metabolic state of bacteria (50, 51). Bacteriostatic antibiotics have been shown to slow bacterial respiration, but bactericidal antibiotics increase bacterial respiration (52, 53). Although carbadox is a bactericidal antibiotic (54) (likely because of phage-mediated lysis), its effects on bacterial metabolism resemble those of a bacteriostatic antibiotic; carbadox induced significant downregulation of pathways related to cell growth and division (i.e., cell wall and capsule, regulation and cell signaling, and RNA metabolism) and potential energy-yielding pathways (i.e., carbohydrates, respiration, amino acid metabolism, protein metabolism, DNA metabolism, and metabolism of aromatic compounds). These shifts probably occurred because of a combination of carbadox-induced changes in metabolic pathway gene expression and bacteriostatic and bactericidal effects on both aerobic and anaerobic populations. These perturbations occur on the same day as population shifts observed in the 16S rRNA gene sequence data (22) and in the phage induction pathways. The perturbation is further manifested by downregulation of central cellular processes, amino acid cycling, RNA metabolism, and cofactor biosynthesis. This abrupt microbiome perturbation eventually establishes a new stable state by 1 week after antibiotic introduction. These perturbations may result in excess high-energy metabolites (as has been observed under other conditions [55]) (i.e., pyruvate, amino acids, nucleotides) in the intestinal lumen, which may supply energy to the host. In the future, it will be critical to study the metabolite composition of the gut because of dietary perturbations to enable a causative connection between the microbiota and the host response and an antibiotic’s mechanism as an animal growth-promoting feed additive.

### Conclusions.

This study shows that in-feed carbadox promoted the expression of prophage genes relevant to all steps of the phage lytic cycle. Phage DNA obtained from the same fecal samples contained antibiotic resistance genes and, in one case, multiple antibiotic resistance genes in series. None of these resistance genes are known to confer resistance to carbadox; thus, phages may mobilize genes that confer resistance to a suite of antibiotics beyond those administered to swine in the present study. Bacterial phage response and defense genes are underexpressed with carbadox, while phage integrase genes are overexpressed, indicating that transduction is an avenue of horizontal gene transfer that is likely made more active in the swine gut by the use of therapeutic doses of carbadox. Although the relative abundance of most antibiotic resistance genes was not enriched in the viromes of animals that received carbadox, we expect greater mobilization of genetic material in animals receiving carbadox because of the increase in the expression of lytic phage genes. Thus, phage-mediated effects on the microbiome are important collateral effects of antibiotics. In future studies, phage and prophage DNA in bacterial communities should be sequenced to determine the identities of the genes transferred, the phage host range, and the impact of lytic phage on the composition of the bacterial community.

## MATERIALS AND METHODS

### Sample collection.

DNA and RNA were extracted from fecal samples from piglets (*n* = 12) from two sows that were divided into two treatment groups (nonamended feed or 50 g of carbadox/ton of feed) as described in a previous publication describing the analysis of 16S rRNA gene sequences from these samples (22). Briefly, microbial metatranscriptome and metagenome libraries were made from samples collected 5 days prior to and 1, 2, and 7 days following carbadox initiation in feed, while virome libraries were made for samples collected 5 days prior to and 2 and 7 days following carbadox initiation in feed. One animal could not be sampled at the day 1 time point, so there were 47 samples for metatranscriptome and metagenome libraries and 36 samples for virome libraries. Prior to nucleic acid extraction, the fecal material was spiked with artificial RNA (a 1,008-bp RNA fragment purified from SmaI digestion of expression products from pGEMEx-1; 250 mg of feces spiked with 17 ng [4.1 × 10^10^ copies] of RNA as described previously [56]) to allow normalization of transcript counts by grams of fecal material. Metatranscriptome and microbial metagenome libraries were prepared by extraction of RNA and DNA from a single tube of fecal material with the PowerMag Microbiome RNA/DNA isolation kit in accordance with the manufacturer’s instructions for RNA and DNA isolation (Mo Bio Laboratories, Inc., Solana Beach, CA). rRNA was removed with the Metabacteria Ribo-Zero kit (Illumina). RNA was converted to cDNA, and sequencing libraries were constructed with the TruSeq RNA kit V2 (Illumina). Double-stranded DNA virions were isolated by CsCl gradient ultracentrifugation size selection, followed by DNase treatment to eliminate exogenous DNA prior to virion lysis as described previously (20) and on the basis of previously published protocols (57) (see Text S1 for a detailed protocol description) to create virome libraries.

### Sequencing and quality filtering.

Microbial community RNA was sequenced with Illumina HiSeq2000 (1 × 100 bp; Iowa State University, Ames, IA) in two eight-lane flow cells in accordance with the manufacturer’s instructions. Microbial community DNA was sequenced on a Roche GS-FLX instrument with two FLX plus chemistry (454 Life Sciences) plates, and phage DNA was sequenced on a Roche GS-FLX instrument with one titanium chemistry plate. The microbial metatranscriptome, microbial metagenome, and virome were all used to form a composite assembly, and an assembly of the phage sequences alone was also completed. ORFs in contigs were predicted, sequences were mapped to ORFs and counted (Table S1), and ORFs were annotated by using the FIGfams and SEED subsystems databases (58), as well as the Resfams (59) and minCED (60) tools. The viral sequences were uploaded to VIROME (61) for annotation. Sequence analysis is described in more detail in Text S1.

10.1128/mBio.00709-17.8 TABLE S1  Sequence counts, filtering, spiked-in RNA counts, and mapping statistics obtained from metatranscriptomic sequences. Download TABLE S1, XLSX file, 0.1 MB. Copyright © 2017 Johnson et al. 2017 Johnson et al. This content is distributed under the terms of the Creative Commons Attribution 4.0 International license.

10.1128/mBio.00709-17.9 TABLE S2  Statistics of the composite assembly (metatranscriptome, microbial metagenome, and virome) and the virome assembly. Download TABLE S2, XLSX file, 0.04 MB. Copyright © 2017 Johnson et al. 2017 Johnson et al. This content is distributed under the terms of the Creative Commons Attribution 4.0 International license.

10.1128/mBio.00709-17.10 TABLE S3  Resistance genes identified in the virome assembly with the Resfams database. Download TABLE S3, TXT file, 0.01 MB. Copyright © 2017 Johnson et al. 2017 Johnson et al. This content is distributed under the terms of the Creative Commons Attribution 4.0 International license.

### Statistics.

To normalize between samples, an artificial RNA fragment was used to spike the fecal samples and sequenced with the microbial community metatranscriptome. Raw counts of the known spiked-in RNA in each sample were determined by aligning the raw read data with the known spiked-in gene reference sequence by using GSNAP. RNA count tables were multiplied by the median raw counts of spiked-in RNA in all of the samples divided by the spiked-in RNA counts of individual samples to normalize the counts to the weight of the original fecal samples. Statistics were all completed with counts normalized to spiked-in RNA counts. QuasiSeq v. 1.0-8, an R bioconductor package, was used to compare samples treated with carbadox-treated and untreated samples. The statistical model used normalized read counts as the dependent variable. A negative binomial distribution of read count data was assumed. A spline correction was implemented within QuasiSeq to account for some of the overdispersion in the count data. Multiple-testing correction was completed by using a false discovery rate correction, the output of which is a *q* value (62). NMDS with Bray-Curtis diversity as the measure of beta diversity was performed in R with the vegan package (version 2.3-5) (63). Phage counts were subsampled to an even depth (4,788 sequences), and comparisons were of relative abundance.

### Accession number(s).

The sequence data obtained in this study have been deposited in the NCBI Sequence Read Archive under accession numbers SRX2664029 to SRX2664164 in association with BioProject PRJNA237795.

## References

[1] Federal Register 2015 Veterinary feed directive. Fed Regist 80:31707–31735. https://www.federalregister.gov/documents/2015/06/03/2015-13393/veterinary-feed-directive.

[2] Stokes HW, Gillings MR 2011 Gene flow, mobile genetic elements and the recruitment of antibiotic resistance genes into Gram-negative pathogens. FEMS Microbiol Rev 35:790–819. doi:10.1111/j.1574-6976.2011.00273.x. 21517914

[3] Centers for Disease Control and Prevention 2013 Antibiotic resistance threats in the United States, 2013. Centers for Disease Control and Prevention, Atlanta, GA https://www.cdc.gov/drugresistance/pdf/ar-threats-2013-508.pdf.

[4] Andersson DI, Hughes D 2014 Microbiological effects of sublethal levels of antibiotics. Nat Rev Microbiol 12:465–478. doi:10.1038/nrmicro3270. 24861036

[5] Davies J, Spiegelman GB, Yim G 2006 The world of subinhibitory antibiotic concentrations. Curr Opin Microbiol 9:445–453. doi:10.1016/j.mib.2006.08.006. 16942902

[6] Pérez-Cobas AE, Gosalbes MJ, Friedrichs A, Knecht H, Artacho A, Eismann K, Otto W, Rojo D, Bargiela R, von Bergen M, Neulinger SC, Däumer C, Heinsen FA, Latorre A, Barbas C, Seifert J, dos Santos VM, Ott SJ, Ferrer M, Moya A 2013 Gut microbiota disturbance during antibiotic therapy: a multi-omic approach. Gut 62:1591–1601. doi:10.1136/gutjnl-2012-303184. 23236009 PMC3812899

[7] Paterson DL 2004 “Collateral damage” from cephalosporin or quinolone antibiotic therapy. Clin Infect Dis 38(Suppl 4):S341–SS345. doi:10.1086/382690. 15127367

[8] Looft T, Johnson TA, Allen HK, Bayles DO, Alt DP, Stedtfeld RD, Sul WJ, Stedtfeld TM, Chai B, Cole JR, Hashsham SA, Tiedje JM, Stanton TB 2012 In-feed antibiotic effects on the swine intestinal microbiome. Proc Natl Acad Sci U S A 109:1691–1696. doi:10.1073/pnas.1120238109. 22307632 PMC3277147

[9] Munk P, Andersen VD, de Knegt L, Jensen MS, Knudsen BE, Lukjancenko O, Mordhorst H, Clasen J, Agersø Y, Folkesson A, Pamp SJ, Vigre H, Aarestrup FM 2017 A sampling and metagenomic sequencing-based methodology for monitoring antimicrobial resistance in swine herds. J Antimicrob Chemother 72:385–392. doi:10.1093/jac/dkw415. 28115502

[10] Zhu YG, Johnson TA, Su JQ, Qiao M, Guo GX, Stedtfeld RD, Hashsham SA, Tiedje JM 2013 Diverse and abundant antibiotic resistance genes in Chinese swine farms. Proc Natl Acad Sci U S A 110:3435–3440. doi:10.1073/pnas.1222743110. 23401528 PMC3587239

[11] Salyers AA, Shoemaker NB, Li LY 1995 In the driver’s seat: the *Bacteroides* conjugative transposons and the elements they mobilize. J Bacteriol 177:5727–5731. doi:10.1128/jb.177.20.5727-5731.1995. 7592315 PMC177390

[12] USDA 2007 Swine 2006, part II: reference of swine health and health management practices in the United States, 2006. USDA, Fort Collins, CO https://www.aphis.usda.gov/animal_health/nahms/swine/downloads/swine2006/Swine2006_dr_PartII.pdf.

[13] Chen Q, Tang S, Jin X, Zou J, Chen K, Zhang T, Xiao X 2009 Investigation of the genotoxicity of quinocetone, carbadox and olaquindox in vitro using Vero cells. Food Chem Toxicol 47:328–334. doi:10.1016/j.fct.2008.11.020. 19061932

[14] Beutin L, Preller E, Kowalski B 1981 Mutagenicity of quindoxin, its metabolites, and two substituted quinoxaline-di-*N*-oxides. Antimicrob Agents Chemother 20:336–343. doi:10.1128/AAC.20.3.336. 7030199 PMC181697

[15] Mersch-Sundermann V, Schneider U, Klopman G, Rosenkranz HS 1994 SOS induction in *Escherichia coli* and *Salmonella* mutagenicity: a comparison using 330 compounds. Mutagenesis 9:205–224. doi:10.1093/mutage/9.3.205. 7934961

[16] Bearson BL, Allen HK, Brunelle BW, Lee IS, Casjens SR, Stanton TB 2014 The agricultural antibiotic carbadox induces phage-mediated gene transfer in *Salmonella*. Front Microbiol 5:52. doi:10.3389/fmicb.2014.00052. 24575089 PMC3920066

[17] Little JW, Mount DW 1982 The SOS regulatory system of *Escherichia coli*. Cell 29:11–22. doi:10.1016/0092-8674(82)90085-X. 7049397

[18] Stanton TB, Humphrey SB, Sharma VK, Zuerner RL 2008 Collateral effects of antibiotics: carbadox and metronidazole induce VSH-1 and facilitate gene transfer among *Brachyspira hyodysenteriae* strains. Appl Environ Microbiol 74:2950–2956. doi:10.1128/AEM.00189-08. 18359835 PMC2394957

[19] Touchon M, Bernheim A, Rocha EP 2016 Genetic and life-history traits associated with the distribution of prophages in bacteria. ISME J 10:2744–2754. doi:10.1038/ismej.2016.47. 27015004 PMC5113838

[20] Allen HK, Looft T, Bayles DO, Humphrey S, Levine UY, Alt D, Stanton TB 2011 Antibiotics in feed induce prophages in swine fecal microbiomes. mBio 2:00260-11. doi:10.1128/mBio.00260-11. PMC3225969 22128350

[21] Darwin AJ 2005 The phage-shock-protein response. Mol Microbiol 57:621–628. doi:10.1111/j.1365-2958.2005.04694.x. 16045608

[22] Looft T, Allen HK, Casey TA, Alt DP, Stanton TB 2014 Carbadox has both temporary and lasting effects on the swine gut microbiota. Front Microbiol 5:276. doi:10.3389/fmicb.2014.00276. 24959163 PMC4050737

[23] Hansen LH, Johannesen E, Burmølle M, Sørensen AH, Sørensen SJ 2004 Plasmid-encoded multidrug efflux pump conferring resistance to olaquindox in *Escherichia coli*. Antimicrob Agents Chemother 48:3332–3337. doi:10.1128/AAC.48.9.3332-3337.2004. 15328093 PMC514751

[24] Codex Alimenarius Commission 2013 Report of the 21st session of the Codex Committee on Residues of Veterinary Drugs in Foods. World Health Organization, Geneva, Switzerland http://www.fao.org/input/download/report/802/REP14_RVe.pdf.

[25] Howe A, Ringus DL, Williams RJ, Choo ZN, Greenwald SM, Owens SM, Coleman ML, Meyer F, Chang EB 2016 Divergent responses of viral and bacterial communities in the gut microbiome to dietary disturbances in mice. ISME J 10:1217–1227. doi:10.1038/ismej.2015.183. 26473721 PMC5029215

[26] Schubert RA, Dodd IB, Egan JB, Shearwin KE 2007 Cro’s role in the CI-Cro bistable switch is critical for lambda’s transition from lysogeny to lytic development. Genes Dev 21:2461–2472. doi:10.1101/gad.1584907. 17908932 PMC1993876

[27] Lemire S, Figueroa-Bossi N, Bossi L 2011 Bacteriophage crosstalk: coordination of prophage induction by *trans*-acting antirepressors. PLoS Genet 7:e1002149. doi:10.1371/journal.pgen.1002149. 21731505 PMC3121763

[28] Bedford E, Tabor S, Richardson CC 1997 The thioredoxin binding domain of bacteriophage T7 DNA polymerase confers processivity on *Escherichia coli* DNA polymerase I. Proc Natl Acad Sci U S A 94:479–484. doi:10.1073/pnas.94.2.479. 9012809 PMC19538

[29] Aksyuk AA, Rossmann MG 2011 Bacteriophage assembly. Viruses 3:172–203. doi:10.3390/v3030172. 21994726 PMC3185693

[30] Botstein D, Waddell CH, King J 1973 Mechanism of head assembly and DNA encapsulation in *Salmonella* phage p22. I. Genes, proteins, structures and DNA maturation. J Mol Biol 80:669–695. doi:10.1016/0022-2836(73)90204-0. 4773026

[31] Isidro A, Henriques AO, Tavares P 2004 The portal protein plays essential roles at different steps of the SPP1 DNA packaging process. Virology 322:253–263. doi:10.1016/j.virol.2004.02.012. 15110523

[32] Trudil D 2015 Phage lytic enzymes: a history. Virol Sin 30:26–32. doi:10.1007/s12250-014-3549-0. 25662888 PMC8200920

[33] Poteete AR, Fenton AC, Murphy KC 1999 Roles of RuvC and RecG in phage lambda red-mediated recombination. J Bacteriol 181:5402–5408. 10464213 10.1128/jb.181.17.5402-5408.1999 PMC94048

[34] Hare JM, Ferrell JC, Witkowski TA, Grice AN 2014 Prophage induction and differential RecA and UmuDAb transcriptome regulation in the DNA damage responses of *Acinetobacter baumannii* and *Acinetobacter baylyi*. PLoS One 9:e93861. doi:10.1371/journal.pone.0093861. 24709747 PMC3978071

[35] Leskinen K, Blasdel BG, Lavigne R, Skurnik M 2016 RNA-sequencing reveals the progression of phage-host interactions between φR1-37 and *Yersinia enterocolitica*. Viruses 8:111. doi:10.3390/v8040111. 27110815 PMC4848604

[36] Weiner L, Brissette JL, Model P 1991 Stress-induced expression of the *Escherichia coli* phage shock protein operon is dependent on sigma 54 and modulated by positive and negative feedback mechanisms. Genes Dev 5:1912–1923. doi:10.1101/gad.5.10.1912. 1717346

[37] Martínez JL, Coque TM, Baquero F 2015 What is a resistance gene? Ranking risk in resistomes. Nat Rev Microbiol 13:116–123. doi:10.1038/nrmicro3399. 25534811

[38] Enault F, Briet A, Bouteille L, Roux S, Sullivan MB, Petit MA 2017 Phages rarely encode antibiotic resistance genes: a cautionary tale for virome analyses. ISME J 11:237–247. doi:10.1038/ismej.2016.90. 27326545 PMC5315482

[39] Kleiner M, Hooper LV, Duerkop BA 2015 Evaluation of methods to purify virus-like particles for metagenomic sequencing of intestinal viromes. BMC Genomics 16:7. doi:10.1186/s12864-014-1207-4. 25608871 PMC4308010

[40] Witkin EM 1976 Ultraviolet mutagenesis and inducible DNA repair in *Escherichia coli*. Bacteriol Rev 40:869–907. 795416 10.1128/br.40.4.869-907.1976 PMC413988

[41] Khil PP, Camerini-Otero RD 2002 Over 1000 genes are involved in the DNA damage response of *Escherichia coli*. Mol Microbiol 44:89–105. doi:10.1046/j.1365-2958.2002.02878.x. 11967071

[42] Courcelle J, Khodursky A, Peter B, Brown PO, Hanawalt PC 2001 Comparative gene expression profiles following UV exposure in wild-type and SOS-deficient *Escherichia coli*. Genetics 158:41–64. 11333217 10.1093/genetics/158.1.41 PMC1461638

[43] Hare JM, Bradley JA, Lin CL, Elam TJ 2012 Diverse responses to UV light exposure in *Acinetobacter* include the capacity for DNA damage-induced mutagenesis in the opportunistic pathogens *Acinetobacter baumannii* and *Acinetobacter ursingii*. Microbiology 158:601–611. doi:10.1099/mic.0.054668-0. 22117008 PMC3352118

[44] Giacomoni PU 1982 Induction by mitomycin C of recA protein synthesis in bacteria and spheroplasts. J Biol Chem 257:14932–14936. 6816801

[45] Love PE, Yasbin RE 1984 Genetic characterization of the inducible SOS-like system of *Bacillus subtilis*. J Bacteriol 160:910–920. 6438063 10.1128/jb.160.3.910-920.1984 PMC215796

[46] Nanda AM, Heyer A, Krämer C, Grünberger A, Kohlheyer D, Frunzke J 2014 Analysis of SOS-induced spontaneous prophage induction in *Corynebacterium glutamicum* at the single-cell level. J Bacteriol 196:180–188. doi:10.1128/JB.01018-13. 24163339 PMC3911129

[47] Witkin EM 1975 Elevated mutability of polA derivatives of *Escherichia coli* B/r at sublethal doses of ultraviolet light: evidence for an inducible error-prone repair system (“SOS repair”) and its anomalous expression in these strains. Genetics 79(Suppl):199–213. 1097302

[48] Poroyko V, White JR, Wang M, Donovan S, Alverdy J, Liu DC, Morowitz MJ 2010 Gut microbial gene expression in mother-fed and formula-fed piglets. PLoS One 5:e12459. doi:10.1371/journal.pone.0012459. 20805981 PMC2929194

[49] Versluis D, D’Andrea MM, Ramiro Garcia J, Leimena MM, Hugenholtz F, Zhang J, Öztürk B, Nylund L, Sipkema D, van Schaik W, de Vos WM, Kleerebezem M, Smidt H, van Passel MWJ 2015 Mining microbial metatranscriptomes for expression of antibiotic resistance genes under natural conditions. Sci Rep 5:11981. doi:10.1038/srep11981. 26153129 PMC4495384

[50] Dwyer DJ, Collins JJ, Walker GC 2015 Unraveling the physiological complexities of antibiotic lethality. Annu Rev Pharmacol Toxicol 55:313–332. doi:10.1146/annurev-pharmtox-010814-124712. 25251995

[51] Newton DF, Macfarlane S, Macfarlane GT 2013 Effects of antibiotics on bacterial species composition and metabolic activities in chemostats containing defined populations of human gut microorganisms. Antimicrob Agents Chemother 57:2016–2025. doi:10.1128/AAC.00079-13. 23403424 PMC3632929

[52] Lobritz MA, Belenky P, Porter CBM, Gutierrez A, Yang JH, Schwarz EG, Dwyer DJ, Khalil AS, Collins JJ 2015 Antibiotic efficacy is linked to bacterial cellular respiration. Proc Natl Acad Sci U S A 112:8173–8180. doi:10.1073/pnas.1509743112. 26100898 PMC4500273

[53] Dwyer DJ, Belenky PA, Yang JH, MacDonald IC, Martell JD, Takahashi N, Chan CTY, Lobritz MA, Braff D, Schwarz EG, Ye JD, Pati M, Vercruysse M, Ralifo PS, Allison KR, Khalil AS, Ting AY, Walker GC, Collins JJ 2014 Antibiotics induce redox-related physiological alterations as part of their lethality. Proc Natl Acad Sci U S A: 111:E2100–E2109. doi:10.1073/pnas.1401876111. PMC4034191 24803433

[54] Constable PD, Hinchcliff KW, Done SH, Gruenberg W (ed), 2017 Veterinary medicine: a textbook of the diseases of cattle, horses, sheep, pigs and goats, 11th ed, vol 1, p 153–174. Elsevier, Ltd., St. Louis, MO.

[55] Belenky P, Ye JD, Porter CBM, Cohen NR, Lobritz MA, Ferrante T, Jain S, Korry BJ, Schwarz EG, Walker GC, Collins JJ 2015 Bactericidal antibiotics induce toxic metabolic perturbations that lead to cellular damage. Cell Rep 13:968–980. doi:10.1016/j.celrep.2015.09.059. 26565910 PMC4648786

[56] Gifford SM, Sharma S, Rinta-Kanto JM, Moran MA 2011 Quantitative analysis of a deeply sequenced marine microbial metatranscriptome. ISME J 5:461–472. doi:10.1038/ismej.2010.141. 20844569 PMC3105723

[57] Thurber RV, Haynes M, Breitbart M, Wegley L, Rohwer F 2009 Laboratory procedures to generate viral metagenomes. Nat Protoc 4:470–483. doi:10.1038/nprot.2009.10. 19300441

[58] Meyer F, Overbeek R, Rodriguez A 2009 FIGfams: yet another set of protein families. Nucleic Acids Res 37:6643–6654. doi:10.1093/nar/gkp698. 19762480 PMC2777423

[59] Gibson MK, Forsberg KJ, Dantas G 2015 Improved annotation of antibiotic resistance determinants reveals microbial resistomes cluster by ecology. ISME J 9:207–216. doi:10.1038/ismej.2014.106. 25003965 PMC4274418

[60] Bland C, Ramsey TL, Sabree F, Lowe M, Brown K, Kyrpides NC, Hugenholtz P 2007 CRISPR recognition tool (CRT): a tool for automatic detection of clustered regularly interspaced palindromic repeats. BMC Bioinformatics 8:209. doi:10.1186/1471-2105-8-209. 17577412 PMC1924867

[61] Wommack KE, Bhavsar J, Polson SW, Chen J, Dumas M, Srinivasiah S, Furman M, Jamindar S, Nasko DJ 2012 VIROME: a standard operating procedure for analysis of viral metagenome sequences. Stand Genomic Sci 6:427–439. doi:10.4056/sigs.2945050. 23407591 PMC3558967

[62] Storey JD, Tibshirani R 2003 Statistical significance for genomewide studies. Proc Natl Acad Sci U S A 100:9440–9445. doi:10.1073/pnas.1530509100. 12883005 PMC170937

[63] Oksanen J, Blanchet FG, Friendly M, Kindt R, Legendre P, McGlinn D, Minchin PR, O’Hara RB, Simpson GL, Solymos P, Stevens MHH, Szoecs E, Wagner H 2016 Vegan: community ecology package. R package version 2.3-5. R Foundation, Vienna, Austria https://rdrr.io/rforge/vegan/.

