# Supplementary figures and images for: The In-Feed Antibiotic Carbadox Induces Phage Gene Transcription in the Swine Gut Microbiome
Source: mBio. 2017 Aug 8;8(4):e00709-17. doi: 10.1128/mBio.00709-17 (PMC5550749; doi:10.1128/mBio.00709-17)

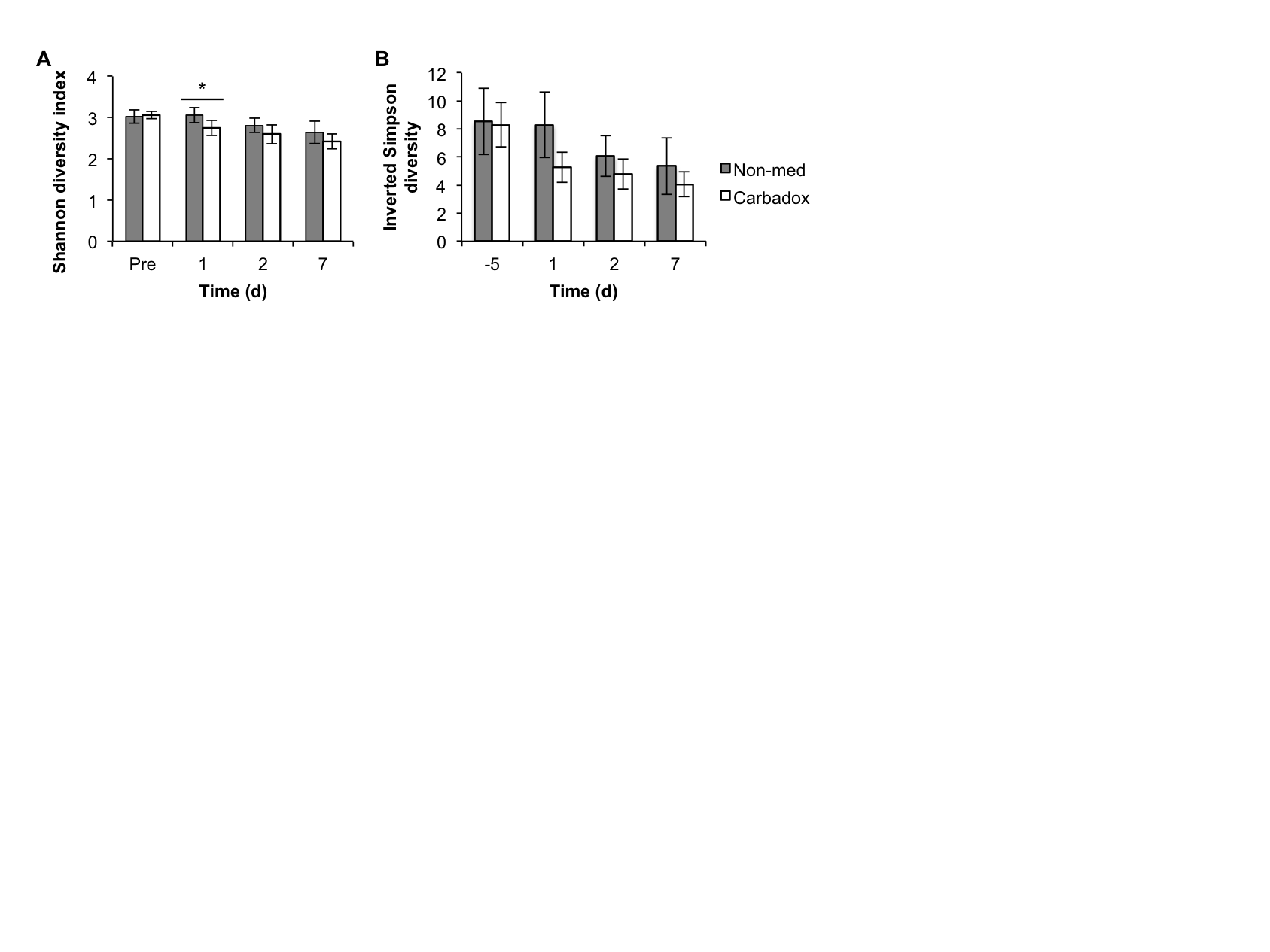

Supplement: FIG S1 [file mbo004173409sf1.tif]

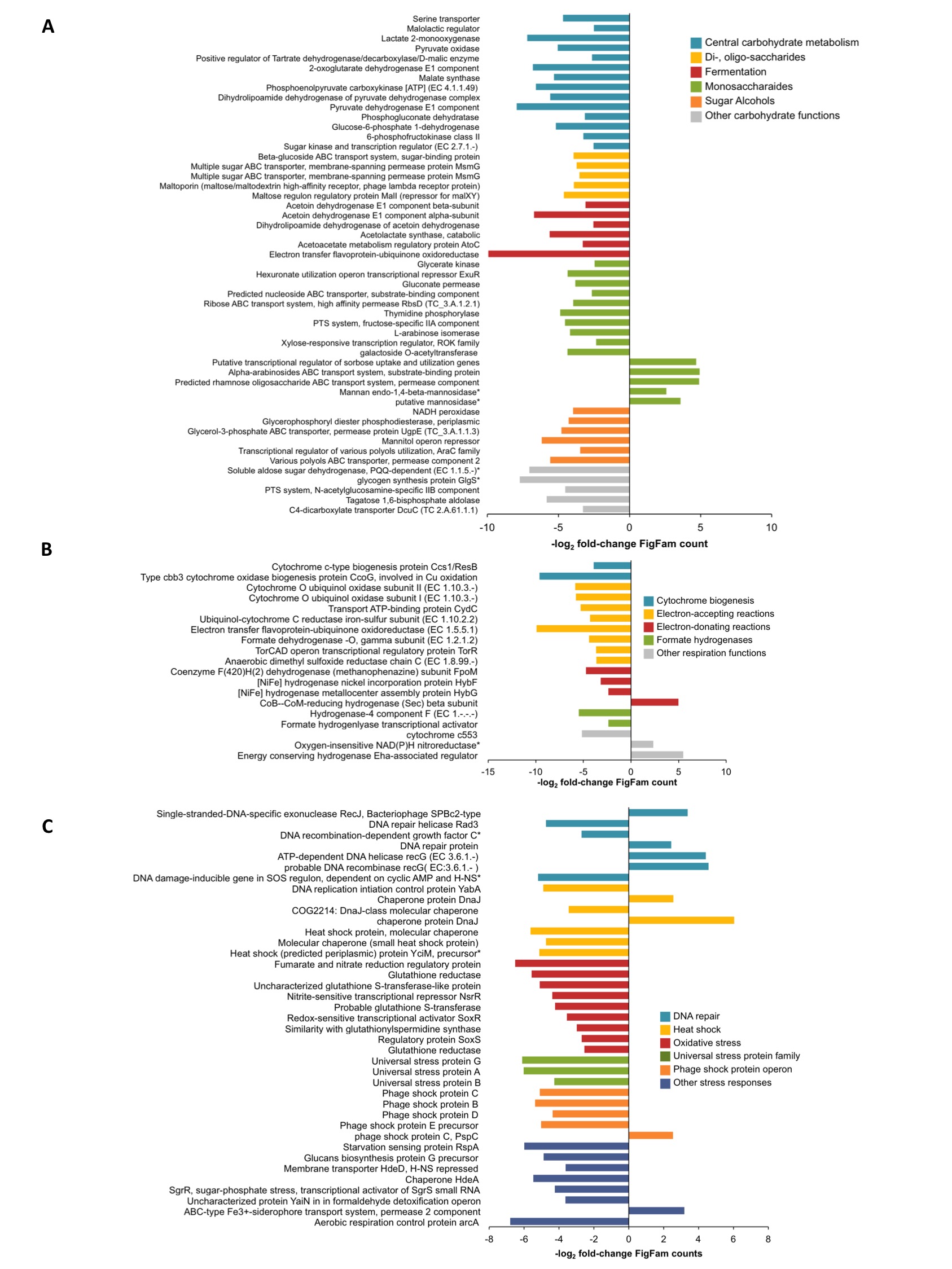

Supplement: FIG S2 [file mbo004173409sf2.jpg]

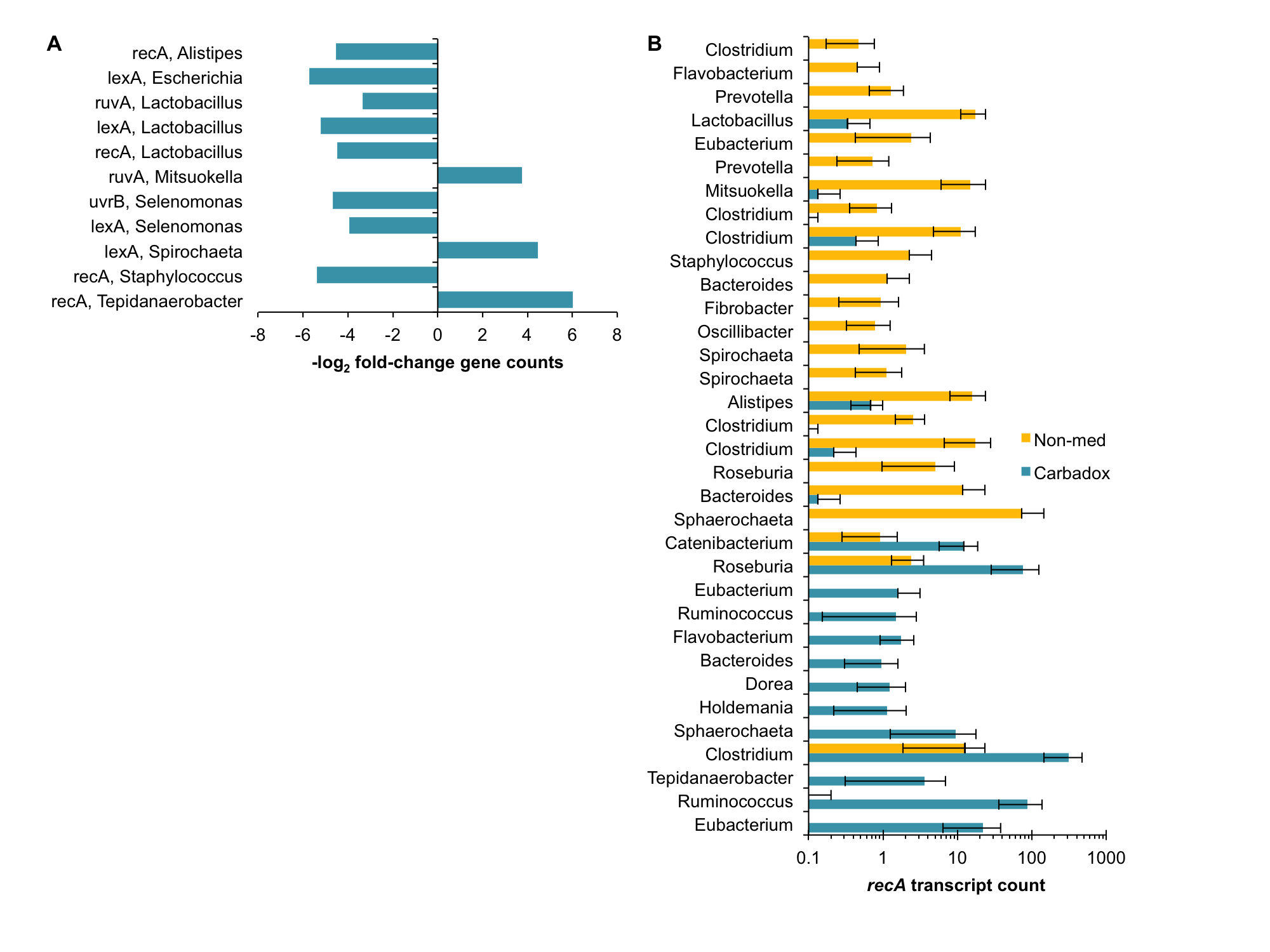

Supplement: FIG S3 [file mbo004173409sf3.tif]

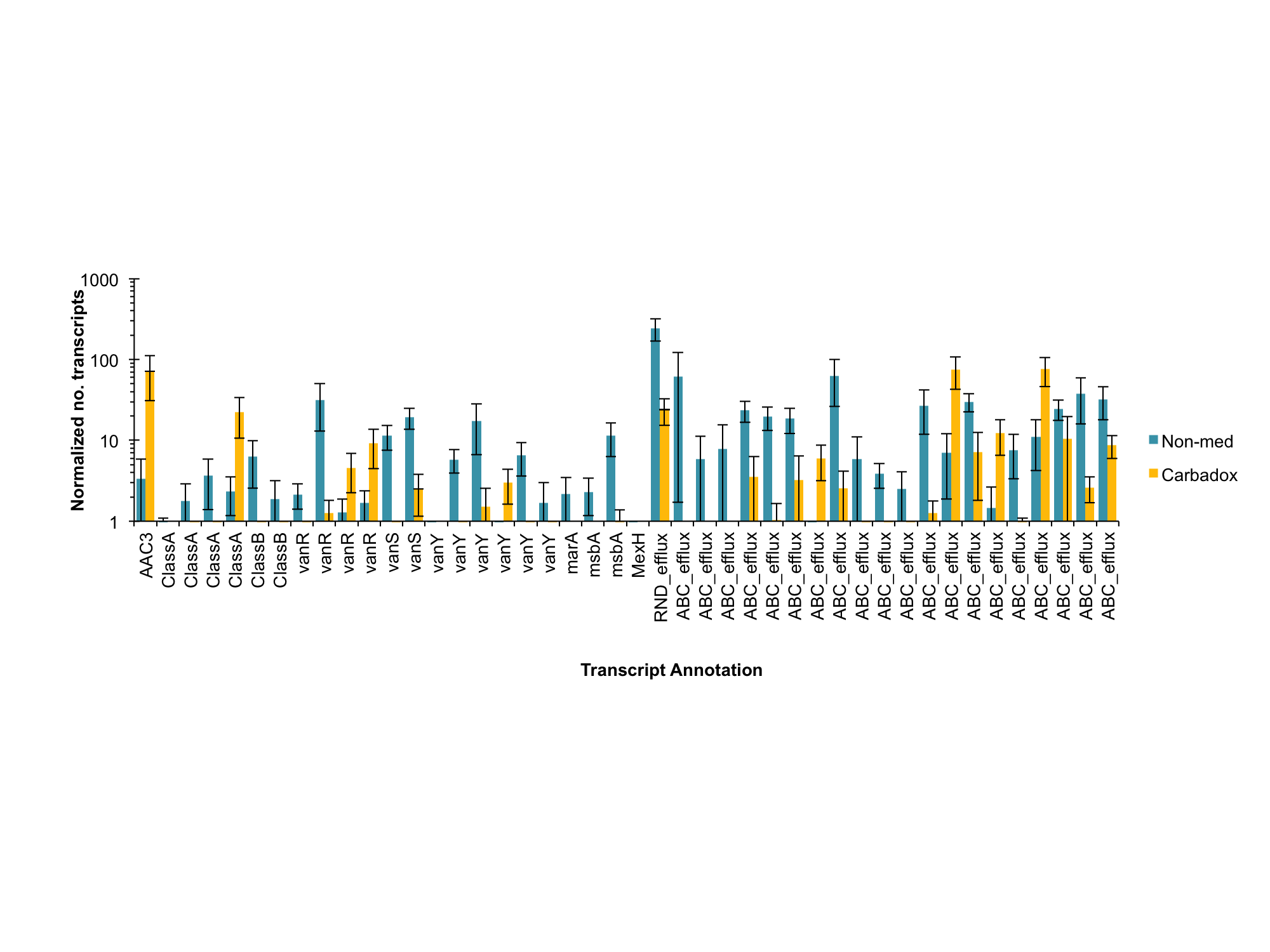

Supplement: FIG S4 [file mbo004173409sf4.tif]

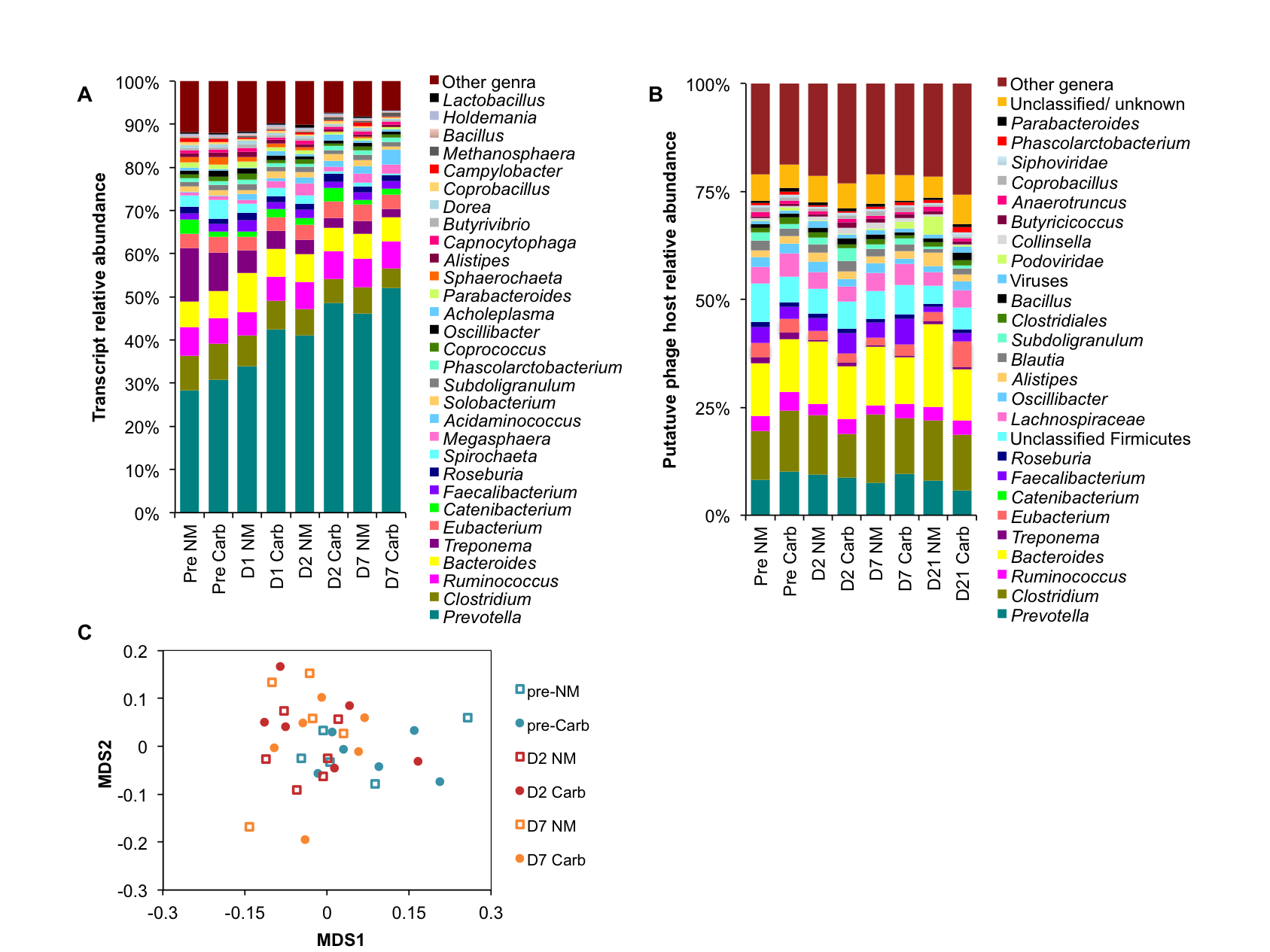

Supplement: FIG S5 [file mbo004173409sf5.tif]

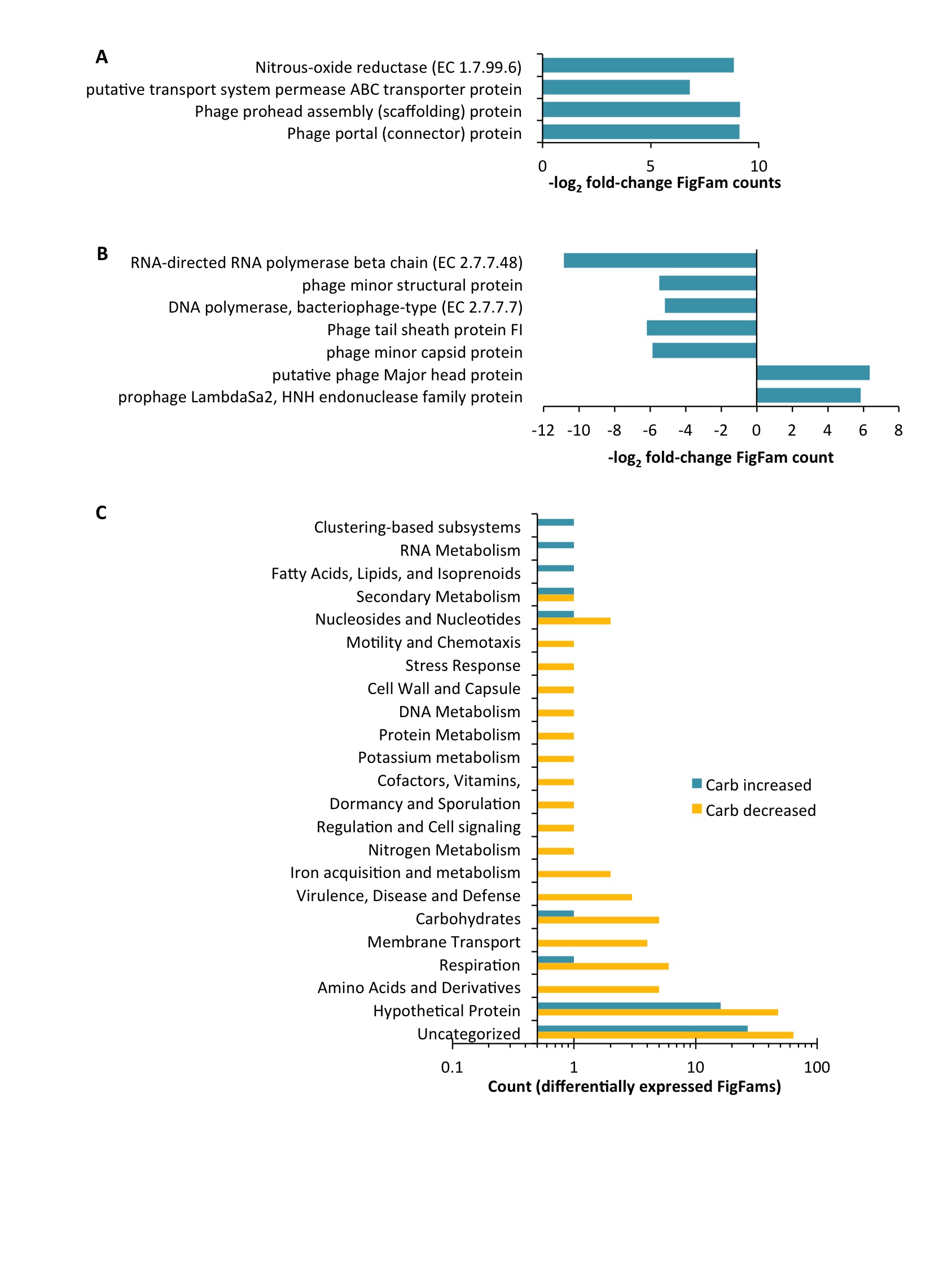

Supplement: FIG S6 [file mbo004173409sf6.jpg]
