# Supplementary material for: The In-Feed Antibiotic Carbadox Induces Phage Gene Transcription in the Swine Gut Microbiome
Source: mBio. 2017 Aug 8;8(4):e00709-17. doi: 10.1128/mBio.00709-17 (PMC5550749; doi:10.1128/mBio.00709-17)
Supplement: TEXT S1 [file mbo004173409s1.docx]

**Supplemental Information:**

**The In-Feed Antibiotic Carbadox Induces Phage Gene Transcription in the Swine Gut Microbiome**

Timothy A. Johnson^1^, Torey Looft^1*^, Andrew J. Severin^2^, Darrell O. Bayles^1^, Daniel J. Nasko^3^, K. Eric Wommack^3^, Adina Howe^4^, and Heather K. Allen^1*^

^1^National Animal Disease Center, Agricultural Research Service, United States Department of Agriculture, Ames, IA 50010, USA

^2^Genome Informatics Facility, Office of Biotechnology, Iowa State University, Ames, IA 50011, USA

^3^Delaware Biotechnology Institute, University of Delaware, Newark, DE 19711, USA

^4^Agricultural and Biosystems Engineering, Iowa State University, Ames, IA 50011, USA

^*^ Corresponding Authors

**Materials and Methods**

*Viral particle isolation*. Virions were isolated as described previously (1). Briefly, 10 g of feces per pig was blended with 50 ml 1 x SM buffer (100 mM NaCl, 8 mM MgSO_4_, 50 nM Tris [pH 7.5], and 0.002% gelatin [wt/vol]). Sample blendate was filtered over nitex mesh (~118-µm pore size; Wildlife Supply, Yulee, FL) and centrifuged three times before loading onto a CsCl_2_ gradient (2). Gradients were ultracentrifuged at 60,000 g for 2 hr at 4°C. Virions were removed from the interface between the 1.35 g/ml and the 1.5 g/ml layers using a 20-gauge needle on a 5 ml syringe. Virions were concentrated on an Ultracel 3K regenerated cellulose concentrator (EMD Millipore, Billerica, Massachusetts). Viral DNAs were then isolated per individual sample as described previously (3). To eliminate free DNAs, in-tact virions were first treated with DNase. The DNase was deactivated and the virions were lysed with formamide. Viral DNA was precipitated, and then extracted by sequential SDS, cetyltrimethylammonium bromide (CTAB), and chloroform treatments. Finally, the clean viral DNA was precipitated, and then evaluated via gel electrophoresis and spectrophotometry.

*Sequence quality filtering*. Artificial replicate metagenomic sequences obtained from the Roche GS-FLX instrument were removed as described previously (4). To remove contaminating rRNA, swine or plant nucleic acids, the metatranscriptome sequences were sequentially aligned (Table S1) to the following databases or genomes with SOAP3-db version 1.4 (5) (arguments: -s 4 -b 2 -L 100) SILVA SSU and LSU rRNA databases (6), Ribosomal Database Project (7) release 10, *Sus scrofa* (GCA_000003025.4), *Glycine max* (GCA_000004515.2), *Zea mays* (GCA_000005005.5), and *Cucumis sativus* (GCA_000004075.2). Adapter and primer sequences were removed from the sequences that passed the initial SOAP3-db filter using Trimmomatic version 0.22 (8). The trimmed transcript sequences were combined and normalized using the khmer software package (9) normalize-by-median.py (10) (arguments: -k 20 -C 20 -N 4 -x 96e9) as previously described in (11) to remove redundant sequences for assembly and reduce computational requirements.

*Assembly and raw read alignment.* Two assemblies were made, one for all the data that would contribute to microbial genomes and a second assembly of sequences from viral particles only. A combined assembly of the filtered metatranscriptome sequences along with the microbial metagenome and virome DNA sequences was produced using RAY version 2.1.0 (12). A kmer value of 41 provided the best assembly based on N50 value and total sequence length of contigs greater than 300 bases. CD-HIT-EST version 4.6 (13) was used to remove any obtained contig that was greater than 99% identical with the following arguments –T 0 –i –c 0.99 –M 0 –mask N. MetaGeneMark version 2.8 (14) was used (arguments: -f G -p 1 -a -d -m MetaGeneMark_v1.mod) with the default model file to identify 946,101 genes and the position of these genes in the 572,839 assembled contigs.

The phage sequences were assembled separately using the Roche gsAssembler version 2.8 incorporating the following refinements.  Contigs were required to > 300 bp, each sequence was constrained to an output in only one contig, contigs were allowed to be extended using the ends of single sequences, and the assembly was run using the “large genome assembly” mode. MetaGeneMark version 3.26 (14) was used (arguments: -f G -p 1 -a -d -m MetaGeneMark_v1.mod) to identify open reading frames in the virome assembly.

Metatranscriptome sequences were aligned to the combined (metagenome/metatranscriptome) assembly using GSNAP (15) version 2013-05-09 with parameters that suppressed soft clipping (--terminal-threshold 100 -i 1 --trim-mismatch-score=0). RNA counts for each gene were determined using HT-Seq version 0.6.1 (16) for the raw sequences that mapped uniquely. Phage sequences were mapped to the phage-only assembly with gmap version 2015-12-31 (17) (arguments: -n 1 --no-chimeras --min-trimmed-coverage=0.97 --min-identity=0.97 -f samse --nofails). HTseq version 0.6.1p1 was used to count phage sequences that mapped uniquely to the phage assembly.

*Sequence annotation.* The obtained open reading frames of the combined assembly were submitted to multiple levels of annotation. MG-RAST tools were used locally to assign open reading frames to a FigFams, and FigFams to a SEED subsystem (18) (svr_assign_using_figfams, svr_roles_to_subsys (downloaded 6/7/2011), Release64, -otu) which resulted in 337,712 ORFs being assigned to one of the 14,427 FigFams detected in our datasets, 4089 FigFams were assigned a SEED subsystem. ORFs assigned to a FigFam without a corresponding SEED subsystem represented a small portion of the number of reads (<10%) and were manually examined by a text search of the FigFam functional description against the SEED database (http://pubseed.theseed.org/, accessed June 2016) cross-referencing the SEED subsystems ontology (http://www.nmpdr.org/FIG/subsys.cgi). Some FigFams could be inferred to be members of a subsystem either due to an updated SEED database, by using annotations that are listed but not yet included in the SEED subsystems classification, or by manual inference of subsystem based on the functional description of the subsystem (e.g. “Phage related lysozyme” as belonging to the Phage subsystem). A FigFam may have been assigned to multiple SEED subsystems due to involvement in multiple pathways, but only unique FigFams within a subsystem were used in the reported counts. CRISPR arrays were predicted in the composite assembly with minCED version 0.2.0 (derived from (19)) requiring at least 3 repeats per array (minNR 3) and counts were obtained using HTSeq. The taxonomic assignment of each open reading frame was obtained by sequence similarity to the SEED database (listed above) requiring at least 75% alignment of the gene sequence to the SEED database and at least 50% identity. The alignment with the highest percent identity was selected as the best taxonomic match. Resistance genes were identified in both the combined assembly and the phage-only assembly using Resfams.hmm version 1.2 models, as described previously (20) implemented with HMMER version 3.1b2 (21) . R scripts used to count sequences within subsystems, quasiseq statistics, and ordinations are provided at https://github.com/john2929/Carbadox.

De-replicated bacteriophage sequences were uploaded to VIROME (22) for SEED category gene functional prediction as well as putative taxonomic assignment. These annotations obtained for the virome dataset was visualized with the R package PhyloSeq v. 1.14.0 to obtain genus counts and to produce non-metric multidimensional scaling (NMDS) plots based on Bray-Curtis distance.

**Results**

*Broad carbadox-mediated impacts on the microbial metatranscriptome.* Central metabolism pathways, especially components of pyruvate metabolism and the pentose phosphate pathway, were down-regulated. The pyruvate dehydrogenase complex, composed of the E1, E2, and E3 components, is the key enzymatic system to convert pyruvate to acetyl-CoA. The pyruvate dehydrogenase E1 and dihyrolipoamide dehydrofenase (E3) components were both down-regulated 207- and 90-fold, respectively. Decreased expression of pyruvate oxidase and phosphoenolpyruvate carboxykinase [ATP] also would impact pyruvate utilization. Some down-regulated genes are involved in the synthesis or regulation of citric acid (TCA) cycle intermediates (2-oxogluterate dehydrogenase, positive regulator of tartrate dehydrogenase, phosphoenolpyruvate carboxykinase [ATP]). Phosphogluconate dehydrogenase, glucose-6-phosphate 1-dehydrogenase and 6-phosphofructokinase class II are all involved in the pentose phosphate pathway. Some fermentative reactions are also down-regulated with carbadox treatment, including the E1 and E3 components of the acetoin dehydrogenase complex that catalyzes the conversion of acetoin and CoA to acetaldehyde and acetyl-CoA, respectively. The only FigFams up-regulated with carbadox are some members of the monosaccharide subsystem, while twice as many monosaccharide subsystem FigFams are down-regulated.

Coupled with the decreased expression of metabolic pathways was the decreased expression of respiration genes (Fig. 2D). Specifically, genes involved in electron-accepting and electron-donating reactions were largely down-regulated, notably those involving cytochromes C and O, which are involved in the electron-transport chain in oxidative phosphorylation and the production of ATP. Half of the differentially expressed genes involved in electron-accepting reactions and all of the differentially expressed genes involved in electron-donating reactions are likely anaerobic pathways. Thus, both anaerobic and aerobic respiratory pathways may be impacted by carbadox treatment, suggesting the metabolic state of the microbiota was significantly affected by carbadox, and their ability to use carbon sources and subsequent energy production was hampered.

Evidence for induction of DNA repair, SOS pathways, or stress response in general was limited (Fig. S2C). For the purpose of this study, we considered FigFams associated with DNA repair as stress-responses due to the DNA intercalating nature of carbadox and that DNA damage typically induces an SOS stress response. Differentially expressed DNA repair FigFams were both up- and down-regulated 2 days post carbadox exposure but are not canonical genes controlled by the SOS pathway. Three DNA repair proteins (*recJ,* *recG* and a “DNA repair protein”) were up-regulated with carbadox, but three were also down-regulated, including a “DNA damage-inducible gene in the SOS regulon”, a first indication that an SOS response is not apparent in the transcriptome dataset. Seven common SOS pathway genes (*recA, lexA, ruvA, ruvB, uvrA, uvrB,* and *uvrC*) and were identified as FigFams in our dataset, but were not statistically differentially expressed due to carbadox (q > 0.28) among all members of the bacterial community. When considering genus-specific SOS genes, no differences were detected in their abundance due to carbadox on day 1 and few differences (mainly down-regulation) on day 2 (Fig. S3A). Some evidence suggested a sub-genus population specific SOS-response (Fig. S3B), but this analysis is problematic due to low sequence counts at this high level of taxonomic resolution. Almost universally, differentially expressed heat shock, oxidative stress, universal stress proteins, phage shock and other stress response proteins are down-regulated, except for a chaperone protein gene *dnaJ*, which may be more involved in binding of RepA to facilitate plasmid DNA replication (23) than stress response.

**References**

1. **Allen HK**, **Looft T**, **Bayles DO**, **Humphrey S**, **Levine UY**, **Alt D**, **Stanton TB**. 2011. Antibiotics in feed induce prophages in swine fecal microbiomes. MBio **2**.

2. **Sambrook J**. 2001. Molecular Cloning: a laboratory manual, 3rd ed.

3. **Thurber RV**, **Haynes M**, **Breitbart M**, **Wegley L**, **Rohwer F**. 2009. Laboratory procedures to generate viral metagenomes. Nat Protoc **4**:470–483.

4. **Gomez-Alvarez V**, **Teal TK**, **Schmidt TM**. 2009. Systematic artifacts in metagenomes from complex microbial communities. ISME J **3**:1314–1317.

5. **Luo R**, **Wong T**, **Zhu J**, **Liu C-M**, **Zhu X**, **Wu E**, **Lee L-K**, **Lin H**, **Zhu W**, **Cheung DW**, **Ting H-F**, **Yiu S-M**, **Peng S**, **Yu C**, **Li Y**, **Li R**, **Lam T-W**. 2013. SOAP3-dp: fast, accurate and sensitive GPU-based short read aligner. PLoS ONE **8**:e65632.

6. **Quast C**, **Pruesse E**, **Yilmaz P**, **Gerken J**, **Schweer T**, **Yarza P**, **Peplies J**, **Glöckner FO**. 2013. The SILVA ribosomal RNA gene database project: improved data processing and web-based tools. Nucleic Acids Res **41**:D590–6.

7. **Cole JR**, **Wang Q**, **Fish JA**, **Chai B**, **McGarrell DM**, **Sun Y**, **Brown CT**, **Porras-Alfaro A**, **Kuske CR**, **Tiedje JM**. 2014. Ribosomal Database Project: data and tools for high throughput rRNA analysis. Nucleic Acids Res **42**:D633–42.

8. **Bolger AM**, **Lohse M**, **Usadel B**. 2014. Trimmomatic: a flexible trimmer for Illumina sequence data. Bioinformatics **30**:2114–2120.

9. **Crusoe MR**, **Alameldin HF**, **Awad S**, **Boucher E**, **Caldwell A**, **Cartwright R**, **Charbonneau A**, **Constantinides B**, **Edvenson G**, **Fay S**, **Fenton J**, **Fenzl T**, **Fish J**, **Garcia-Gutierrez L**, **Garland P**, **Gluck J**, **González I**, **Guermond S**, **Guo J**, **Gupta A**, **Herr JR**, **Howe A**, **Hyer A**, **Härpfer A**, **Irber L**, **Kidd R**, **Lin D**, **Lippi J**, **Mansour T**, **McA'Nulty P**, **McDonald E**, **Mizzi J**, **Murray KD**, **Nahum JR**, **Nanlohy K**, **Nederbragt AJ**, **Ortiz-Zuazaga H**, **Ory J**, **Pell J**, **Pepe-Ranney C**, **Russ ZN**, **Schwarz E**, **Scott C**, **Seaman J**, **Sievert S**, **Simpson J**, **Skennerton CT**, **Spencer J**, **Srinivasan R**, **Standage D**, **Stapleton JA**, **Steinman SR**, **Stein J**, **Taylor B**, **Trimble W**, **Wiencko HL**, **Wright M**, **Wyss B**, **Zhang Q**, **zyme E**, **Brown CT**. 2015. The khmer software package: enabling efficient nucleotide sequence analysis. F1000Res **4**:900. doi: 10.12688/f1000research.6924.1

10. **Brown CT**, **Howe A**, **Zhang Q**. 2012. A reference-free algorithm for computational normalization of shotgun sequencing data. arXiv:1203.4802v2 [q-bio.GN].

11. **Howe AC**, **Jansson JK**, **Malfatti SA**, **Tringe SG**, **Tiedje JM**, **Brown CT**. 2014. Tackling soil diversity with the assembly of large, complex metagenomes. Proc Natl Acad Sci USA **111**:4904–4909.

12. **Boisvert S**, **Laviolette F**, **Corbeil J**. 2010. Ray: simultaneous assembly of reads from a mix of high-throughput sequencing technologies. J Comput Biol **17**:1519–1533.

13. **Fu L**, **Niu B**, **Zhu Z**, **Wu S**, **Li W**. 2012. CD-HIT: accelerated for clustering the next-generation sequencing data. Bioinformatics **28**:3150–3152.

14. **Zhu W**, **Lomsadze A**, **Borodovsky M**. 2010. Ab initio gene identification in metagenomic sequences. Nucleic Acids Res **38**:e132. doi:10.1093/nar/gkq275

15. **Wu TD**, **Nacu S**. 2010. Fast and SNP-tolerant detection of complex variants and splicing in short reads. Bioinformatics **26**:873–881.

16. **Anders S**, **Pyl PT**, **Huber W**. 2015. HTSeq--a Python framework to work with high-throughput sequencing data. Bioinformatics **31**:166–169.

17. **Wu TD**, **Watanabe CK**. 2005. GMAP: a genomic mapping and alignment program for mRNA and EST sequences. Bioinformatics **21**:1859–1875.

18. **Meyer F**, **Overbeek R**, **Rodriguez A**. 2009. FIGfams: yet another set of protein families. Nucleic Acids Res **37**: 6643–6654.

19. **Bland C**, **Ramsey TL**, **Sabree F**, **Lowe M**, **Brown K**, **Kyrpides NC**, **Hugenholtz P**. 2007. CRISPR recognition tool (CRT): a tool for automatic detection of clustered regularly interspaced palindromic repeats. BMC Bioinformatics **8**:209. doi:10.1186/1471-2105-8-209

20. **Gibson MK**, **Forsberg KJ**, **Dantas G**. 2015. Improved annotation of antibiotic resistance determinants reveals microbial resistomes cluster by ecology. ISME J **9**:207–216.

21. **Mistry J**, **Finn RD**, **Eddy SR**, **Bateman A**, **Punta M**. 2013. Challenges in homology search: HMMER3 and convergent evolution of coiled-coil regions. Nucleic Acids Res **41**:e121. doi:10.1093/nar/gkt263

22. **Wommack KE**, **Bhavsar J**, **Polson SW**, **Chen J**, **Dumas M**, **Srinivasiah S**, **Furman M**, **Jamindar S**, **Nasko DJ**. 2012. VIROME: a standard operating procedure for analysis of viral metagenome sequences. Stand Genomic Sci **6**:427–439.

23. **Wickner S**, **Hoskins J**, **McKenney K**. 1991. Function of DnaJ and DnaK as chaperones in origin-specific DNA binding by RepA. Nature **350**:165–167.
